# Density, Speed of Sound, Refractive Index, and the Derived Properties of Binary Mixtures of *N*,*N*-Dimethylacetamide with 1-Butanol, 1-Pentanol, Furfural, or Furfuryl Alcohol at Different Temperatures

**DOI:** 10.1021/acs.jced.4c00275

**Published:** 2024-11-14

**Authors:** Joan Chepkoech Kilele, Amal Ayad, Joseph Saab, Amina Negadi, Ariel Hernández, Indra Bahadur, Vibha Kumar, Mostafizur Rahaman, Latifa Negadi

**Affiliations:** 1 LATA2M, Laboratoire de Thermodynamique Appliquée et Modélisation Moléculaire, University of Tlemcen, Post Office Box 119, Tlemcen 13000, Algeria; 2 Department of Chemistry, Durban University of Technology, Durban 4001, South Africa; 3 Department of Chemistry Biochemistry, Holy Spirit University of Kaslik, Post Office Box 446, Jounieh 446, Lebanon; 4 Departamento de Ingeniería Industrial, Facultad de Ingeniería, Universidad Católica de la Santísima Concepción, Alonso de Ribera 2850, Concepción 4090541,Chile; 5 Department of Chemistry, North-West University (Mafikeng Campus), Private Bag X2046, Mmabatho 2735, South Africa; 6 Department of Chemistry, Pt. L. M. S. Campus, Rishikesh, SDS University, Badshahithau 249201, India; 7 Department of Chemistry, College of Science, King Saud University, P.O. Box 2455, Riyadh 11451, Saudi Arabia; 8 Thermodynamics Research Unit, School of Engineering, University of KwaZulu-Natal, King George V Avenue, Durban 4041, South Africa

## Abstract

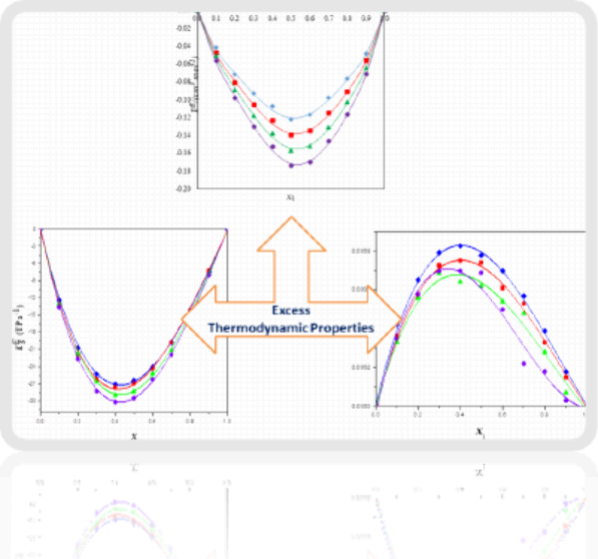

The density (ρ), speed of sound (*c*), and refractive index (*n*_D_) of *N*,*N*-dimethylacetamide (DMA) with 1-butanol, 1-pentanol, furfural (FFL), or furfuryl alcohol (FA) as a function of composition and at *T* = 293.15 to 323.15 K with an interval of 10 K and atmospheric pressure were measured. From the experimental data, the excess molar volume (*V*_m_^E^), isentropic compressibility (*κ*_s_), intermolecular free length (*L*_f_), specific acoustic impedance (*Ζ*), relative association (*R*_A_), relaxation strength (*r*), Rao’s molar sound function (*R*), excess isentropic compressibility (*k*_s_^E^), and excess refractive index (*n*_D_^E^) properties were calculated. These results were successfully fitted to the Redlich–Kister polynomial equation. The obtained results were discussed in terms of the nature of molecular interactions. The perturbed chain statistical associating fluid theory equation of state (PC-SAFT EoS) as a predictive approach was used for modeling the density of the binary mixtures. Schaaffs's collision factor theory (SCFT) and Nomoto’s relation (NR) were successfully applied for predictive modeling the speed of sound of the binary mixtures, and four mixing rules were used for the modeling of the refractive index of the mixtures.

## 1 Introduction

Electrospun carbon nanofibers/membranes prepared via the electrospinning technique mainly using polymer solutions are attractive multifunctional nanomaterials with a huge potential to be applied in different fields such as textile, pharmaceutical, as well as water remediation and air purification.^1,2^ Electrospinning is an industry-sustainable technology that provides straightforward and low-cost fabrication strategies producing composite nanofibers from a number of precursors such as natural and synthetic polymers.^1,3^ This method utilizes solvents such as *N*,*N*-dimethylacetamide, ethylene glycol, and *N*,*N*-dimethyl formamide to dissolve the polymers; hence, the choice of solvents used has a significant influence on the structure of the membrane/nanofibers owing to their different properties.^1,4,5^

A number of work have been reported on the improvement of electrospun nanofibers using different binary or tertiary solvent systems including water/*N,N*-dimethylacetamide,^4^ lithium chloride/*N*,*N*-dimethylacetamide, 1-allyl-3-methylimidazolium chloride/*N*,*N*-dimethylformamide, methyl ethyl ketone and *N*,*N*-dimethyl acetamide,^6^ acetone/dimethylacetamide,^7^ and acetone/water systems. However, the major challenges affecting the performance of these solvent systems are the high volatility of acetone and relatively high costs, and they may lead to the release of harmful byproducts and thus cannot be considered to be sustainable.^1,5^ Based on this fact, researchers have been working on discovering a novel and more suitable alternative binary/tertiary system that is highly efficient and comparatively cost-effective. Reliable information about the thermophysical properties of the chosen system is required to understand the molecular interactions. Experimentally determined physical properties including the density, speed of sound, and viscosity of the mixtures under varying compositions and temperatures have been used extensively to investigate the molecular interactions among the molecular components in the binary or tertiary systems. The study of excess/derived properties of the thermophysical properties of the respective systems provides useful information regarding the strength as well as the nature of the intra/extramolecular interactions in the constituent system.

*N*,*N*-Dimethylacetamide (DMA), a polar aprotic solvent, has received extensive attention owing to its remarkable properties including high boiling point, good thermal stability, low toxicity, as well as miscibility in most organic solvents; hence, it is widely used in many industrial processes.^8^ Recently, a few authors reported on the investigation of the volumetric, acoustic and viscometric properties of the mixtures of DMA as the main component with other organic solvents including alcohols,^8^ ionic liquids,^9^ ketones^10^ and polyethylene glycols^11^ for use in various industrial applications. In spite of the unlimited range of the applications of DMA, very few or no experimental studies have been conducted on the thermodynamic properties of the binary as well as ternary mixtures of DMA (1) + 1-butanol (2), + 1-pentanol (2), + FFL (2), or + FA (2). Therefore, knowledge of their thermodynamic properties is essential for understanding the intermolecular interactions occurring between the components of these systems for potential use in dissolution of polymers in the fabrication of electrospun nanofibers. Reportedly, alcohols including 1-butanol and 1-pentanol are used as exceptional organic solvents for a wide variety of industrial processes and chemical reactions because they are relatively cheap and readily available at high percentage purity.^12^ In addition, they are polar and are self-associated through hydrogen bonding.^13^

Furfural is a promising organic solvent that is used in various industrial processes. It is derived from biomass.^14,15^ Furfuryl alcohol, a renewable key chemical intermediate produced by the hydrogenation of furfural, is widely used as a solvent in the manufacture of a number of industrial products including adhesives, pharmaceuticals, plasticizers, and resins^15,16^ Nduli and Deenadayalu^17^ studied the thermophysical properties of methanol or 1-ethyl-3-methylimidazolium acetate + furfural or furfuryl alcohol at *T* = 298.15, 303.15, 308.15, 313.15, and 318.15 K. Wongsawa et al.^18^ investigated the liquid–liquid equilibrium for the ternary systems of water + furfuryl alcohol + (MIBK, ethyl acetate, furfural, or *n*-butanol) at *T* = 298.2 K and at atmospheric pressure.

Therefore, the aim of the present work is to study the thermodynamic properties—density (ρ), speed of sound (*c*), and refractive index (*n*_D_)—of the DMA (1) + 1-butanol (2), DMA (1) + 1-pentanol (2), DMA (1) + FFL (2), and DMA (1) + FA (2) binary systems as a function of composition and temperature from *T* = 293.15 to 323.15 K at atmospheric pressure. The excess properties such as excess molar volume (*V*_m_^E^), excess isentropic compressibility (*k*_s_^E^), and excess refractive index (*n*_D_^E^) were calculated from the measured experimental data. The specific goals of this study are to understand the intermolecular interactions, predict the behavior, and improve the process design of these mixtures.

Finally, the perturbed chain statistical associating fluid theory (PC-SAFT)^19,20^ was used for the modeling of density for the pure fluids and binary mixtures. Also, PC-SAFT + Schaaffs’s collision factor theory (SCFT)^21^ and PC-SAFT + Nomoto’s relation^22^ were used to compute the speed of sound, and the refractive index for the mixtures was calculated predictively from PC-SAFT + four mixing rules.^23^

## 2 Experimental Methods

### 2.1 Chemicals

The chemicals used in this study together with their basic information are summarized in Table 1. *N*,*N*-Dimethylacetamide (0.99 in mass fraction), 1-pentanol (≥0.99 in mass fraction), furfural (0.99 in mass fraction), and furfuryl alcohol (0.98 in mass fraction) were supplied by Sigma-Aldrich. In addition, 1-butanol (0.999 in mass fraction) was purchased from Honeywell. All of the chemicals were used as received.

**Table 1:** Chemical Specifications as per the Suppliers

**chemical**	**CAS #**	**LOT #**	**supplier**	**molar mass** (g/mol)	**mass fraction purity**^a^	**mass fraction purity**^b^
*N*,*N*-dimethylacetamide (DMA)	127-19-5	BCCG6691	Sigma-Aldrich	87.12	0.99	0.99
1-butanol	71-36-3	K2110	Honeywell	74.12	≥0.999	≥0.998
1-pentanol	71-41-0	BCBR6187	Sigma-Aldrich	88.15	≥0.99	≥0.98
furfural (FFL)	98-01-1	MKCL4343	Sigma-Aldrich	96.08	0.99	0.99
furfuryl alcohol (FA)	98-00-0	MKBP8421V	Sigma-Aldrich	98.10	0.98	0.98

a Stated by the supplier.

b GC analysis.

#### 2.1.1 Density and Speed of Sound

Prior to measurements, the binary mixtures of *N*,*N*-dimethylacetamide (1) + 1-butanol (2), 1-pentanol (2), FFL (2), or FA (2) under study were prepared by weighing the mass (g) of the respective pure compounds into the sample vials with the help of an electronic balance (OHAUS, with an accuracy of 0.0001 g). The uncertainty in the mole fraction was 0.0006. Then the mixtures were agitated to ensure good sample mixing and homogeneity. In addition, each of the mixtures as well as the pure compounds was degassed before injection into the instrument using an ultrasonic bath to obtain a homogeneous and bubble-free solution. The pure compounds (DMA, 1-butanol, 1-pentanol, FFL, and FA) together with their corresponding binary mixtures with defined mole fractions were injected into the instrument using a syringe.

The densities and speeds of sound were measured simultaneously at temperatures from 293.15 to 323.15 K using a digital vibrating U-tube densimeter (Anton Paar DSA 5000 M) with an uncertainty of 0.8 kg·m^–3^, 2.81 m·s^–1^, and 0.02 K for density, speed of sound, and temperature, respectively. The measured data of density and speed of sound were displayed together on the LED display at the end of each measurement. The measuring cell was cleaned after each measurement using a suitable solvent and subsequently dried. The instrument was calibrated using the standard solutions provided by the supplier. The technique utilized by the instrument is a well-known oscillating U-tube. The density and speed of sound analyzer (DSA 5000 M) can measure the speed of sound and density simultaneously.

#### 2.1.2 Refractive Index

Measurements of the refractive indices of pure compounds and of each binary mixture were recorded by a digital refractometer (Model Abbemat 300, Anton Paar) provided with a sodium D-line (wavelength of 589 nm) and a temperature precision of 0.02 K. Calibration was performed each day using distilled water. Prior to each measurement, it was cleaned thoroughly by using acetone. The uncertainty was estimated for the refractive index of 0.0007. The uncertainties in excess molar volume, excess isentropic compressibility, excess refractive index, intermolecular free length, specific acoustic impedance, relative association, relaxation strength, and Rao’s molar sound function were 0.003 cm^3^·mol^–1^, 0.17 TPa^–1^, 0.0008, 0.006 × 10^–12^ m, 0.007 × 10^5^ kg m^–2^ s^–1^, 0.0009, 0.0008, and 0.0007 × 10^–3^ m (10/3) mol^–1^ s^–1/3^.

## 3 Theoretical Modeling of Properties

### 3.1 PC-SAFT

PC-SAFT EoS requires the following parameters: the number of segments (*m*), the segment diameter (σ), the depth of pair potential energy (ε/*k*_B_), the association energy of interaction (ε^AB^/*k*_B_), and the effective volume of interaction (κ^AB^) between site A and B on the molecule. For non-self-associating, . In this work, DMA and FFL were modeled as non-self-associating fluids but with the ability to associate with cross-association with self-associating fluids. Therefore, for DMA, one negative site is assumed on each oxygen and nitrogen atom. For FFL, one negative site on each oxygen atom was considered. In the case of alcohols (1-butanol, 1-pentanol, and FA), a 2B scheme was used, i.e., one positive site on the hydrogen atom and the negative site on the oxygen atom. To consider the induced interaction between DMA and alcohols, we must use the approach proposed by Kleiner and Sadowski.^24^ In summary, the parameters of pure fluids must be fitted to vapor pressure and liquid phase density data, and in the case of mixtures, the binary interaction parameter (*k*_*ij*_ = 0) can be null or fitted (*k*_*ij*_ ≠ 0) to experimental liquid density data. In this work, a fully predictive approach (*k*_*ij*_ = 0) was chosen for the binary mixtures. Because the PC-SAFT equations have been widely described in scientific articles, in this work, it was preferred not to rewrite them, but in case the readers are interested, they can go to the original references.^19,20^

### 3.2 SCFT and NR

In this work, we used two models to calculate the speed of sound of binary mixtures by using a predictive approach. The models used were SCFT^21^ and NR.^22^

Using eq 1, the speed of sound in m/s can be calculated from SCFT:
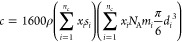
1where *n*_c_ is the number of components, ρ is the molar density for the mixture obtained from PC-SAFT EoS, *s*_*i*_ (*i* = 1, 2, 3) is the space filling factor of fluid *i* in the mixture, *x*_*i*_ is the liquid mole fraction, *N*_A_ represents the Avogadro’s constant, and *d*_*i*_ represents the molecular diameter of component *i*.

On the other hand, using NR, the speed of sound can be obtained from eq 2:
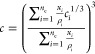
2where ρ_*i*_ and *c*_*i*_ are the density of pure fluid calculated from PC-SAFT EoS and experimental value of speed of sound for the pure fluid, respectively.

### 3.3 Mixing Rules of the Refractive index

In this manuscript, four mixing rules^23^ were used for compute of refractive index, i.e., Laplace (LP), Eykman (EK), Lorentz–Lorenz (LL), and Gladstone–Dale (GD), which are given by following equations, respectively:
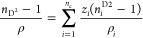
3
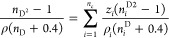
4
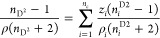
5
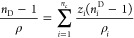
6where *z*_*i*_ and *n*_*Di*_ are the weight fraction of component *i* and the experimental refractive index of pure fluid, respectively. Also, ρ is the liquid density obtained with the PC-SAFT EoS.

## 4 Results and Discussion

In this work, the experimental data of density (ρ), speed of sound (c), and refractive index (n_D_) for the binary mixtures of DMA with 1-butanol, 1-pentanol, FFL, and FA were measured over the entire range of composition at temperatures from *T* = 293.15 to 323.15 K and at atmospheric pressure. The obtained values are listed in Table 2.

**Table 2:** Density (ρ), Speed of Sound (*c*), and Refractive Index (*n*_D_) for the Binary Systems of *N*,*N*-Dimethylacetamide + 1-Butanol, 1-Pentanol, FFL, or FA at Different Temperatures and at Atmospheric Pressure *p* = 0.1 MPa^a^

***x*_1_**	ρ/(kg·m^–3^)	*c/***(m**·**s**^**–1**^**)**	***n*_D_**
DMA + 1-butanol			
*T* = 293.15 K			
0.0000	809.7	1256.75	1.3992
0.0998	823.2	1280.70	1.4036
0.2001	836.6	1303.78	1.4078
0.2997	849.8	1325.68	1.4118
0.3999	863.0	1347.20	1.4157
0.4995	876.0	1368.13	1.4196
0.5998	889.1	1389.28	1.4234
0.7004	902.1	1410.42	1.4272
0.7996	915.0	1431.56	1.4308
0.9004	928.2	1453.50	1.4345
1.0000	941.2	1475.92	1.4382
*T* = 303.15 K			
0.0000	802.1	1222.01	1.3954
0.0998	815.3	1246.18	1.3996
0.2001	828.5	1268.87	1.4037
0.2997	841.5	1290.19	1.4077
0.3999	854.5	1311.07	1.4116
0.4995	867.4	1331.53	1.4154
0.5998	880.4	1352.04	1.4191
0.7004	893.3	1372.56	1.4229
0.7996	906.0	1393.11	1.4264
0.9004	919.1	1414.07	1.4301
1.0000	932.0	1436.08	1.4338
*T* = 313.15 K			
0.0000	794.3	1189.68	1.3914
0.0998	807.4	1212.08	1.3956
0.2001	820.3	1233.71	1.3997
0.2997	833.2	1254.41	1.4036
0.3999	846.0	1275.14	1.4074
0.4995	858.8	1295.03	1.4111
0.5998	871.6	1314.51	1.4149
0.7004	884.4	1334.92	1.4186
0.7996	897.0	1354.89	1.4222
0.9004	909.9	1375.19	1.4258
1.0000	922.7	1396.63	1.4294
*T* = 323.15 K			
0.0000	786.3	1156.62	1.3874
0.0998	799.2	1178.33	1.3915
0.2001	812.1	1199.31	1.3955
0.2997	824.7	1219.81	1.3994
0.3999	837.5	1239.54	1.4033
0.4995	850.1	1258.87	1.4070
0.5998	862.8	1277.82	1.4106
0.7004	875.5	1297.63	1.4144
0.7996	888.0	1317.05	1.4178
0.9004	900.8	1336.83	1.4214
1.0000	913.5	1357.64	1.4251
DMA + 1-pentanol			
*T* = 293.15 K			
0.0000	814.7	1292.08	1.4098
0.0991	825.6	1308.94	1.4125
0.1993	836.8	1325.43	1.4151
0.2994	848.4	1341.94	1.4178
0.4004	860.4	1358.55	1.4205
0.5007	872.8	1375.78	1.4233
0.6000	885.4	1393.32	1.4261
0.7002	898.6	1412.11	1.4289
0.8000	912.3	1431.97	1.4318
0.8994	926.5	1453.45	1.4349
1.0000	941.2	1475.92	1.4382
*T* = 303.15 K			
0.0000	807.4	1258.61	1.4059
0.0991	818.0	1274.84	1.4085
0.1993	829.1	1290.78	1.4111
0.2994	840.4	1306.74	1.4137
0.4004	852.3	1322.77	1.4164
0.5007	864.5	1339.35	1.4191
0.6000	876.9	1356.35	1.4218
0.7002	890.0	1374.52	1.4246
0.8000	903.5	1393.73	1.4275
0.8994	917.4	1414.42	1.4306
1.0000	932.0	1436.08	1.4338
*T* = 313.15 K			
0.0000	799.9	1226.37	1.4021
0.0991	810.4	1240.98	1.4045
0.1993	821.2	1256.39	1.4071
0.2994	832.4	1271.78	1.4097
0.4004	844.1	1287.27	1.4123
0.5007	856.1	1303.35	1.4150
0.6000	868.4	1319.66	1.4176
0.7002	881.3	1337.22	1.4204
0.8000	894.6	1355.74	1.4232
0.8994	908.4	1375.69	1.4262
1.0000	922.7	1396.63	1.4294
*T* = 323.15 K			
0.0000	792.3	1192.41	1.3981
0.0991	802.6	1207.46	1.4005
0.1993	813.3	1222.35	1.4030
0.2994	824.3	1237.21	1.4055
0.4004	835.8	1252.16	1.4081
0.5007	847.7	1267.68	1.4108
0.6000	859.8	1283.43	1.4134
0.7002	872.5	1300.35	1.4161
0.8000	885.6	1318.22	1.4189
0.8994	899.3	1337.46	1.4218
1.0000	913.5	1357.64	1.4251
DMA + FFL			
*T* = 293.15 K			
0.0000	1161.5	1457.83	1.5267
0.1001	1137.7	1459.89	1.5171
0.2002	1114.3	1461.80	1.5078
0.3002	1091.3	1463.88	1.4986
0.3999	1068.8	1465.95	1.4896
0.4999	1046.7	1467.95	1.4807
0.6002	1024.8	1470.22	1.4720
0.6991	1003.5	1471.87	1.4635
0.7998	982.3	1473.85	1.4550
0.9000	961.6	1475.36	1.4465
1.0000	941.2	1475.92	1.4382
*T* = 303.15 K			
0.0000	1150.9	1421.59	1.5215
0.1001	1127.3	1424.05	1.5122
0.2002	1104.0	1425.40	1.5029
0.3002	1081.2	1427.33	1.4938
0.3999	1058.9	1429.15	1.4849
0.4999	1036.9	1430.88	1.4759
0.6002	1015.2	1432.64	1.4673
0.6991	994.0	1433.94	1.4589
0.7998	973.0	1435.81	1.4505
0.9000	952.3	1436.19	1.4420
1.0000	932.0	1436.08	1.4338
*T* = 313.15 K			
0.0000	1140.2	1385.64	1.5163
0.1001	1116.8	1387.53	1.5071
0.2002	1093.7	1389.19	1.4980
0.3002	1071.1	1390.91	1.4890
0.3999	1048.9	1392.52	1.4801
0.4999	1027.1	1393.98	1.4713
0.6002	1005.5	1395.47	1.4627
0.6991	984.5	1396.21	1.4544
0.7998	963.5	1397.07	1.4460
0.9000	943.0	1397.34	1.4377
1.0000	922.7	1396.63	1.4294
*T* = 323.15 K			
0.0000	1129.5	1349.97	1.5111
0.1001	1106.3	1351.71	1.5021
0.2002	1083.4	1353.25	1.4931
0.3002	1060.9	1354.78	1.4841
0.3999	1038.9	1356.18	1.4753
0.4999	1017.3	1357.37	1.4666
0.6002	995.8	1358.48	1.4580
0.6991	974.9	1358.83	1.4498
0.7998	954.1	1359.16	1.4415
0.9000	933.6	1358.93	1.4333
1.0000	913.5	1357.64	1.4251
DMA + FA			
*T* = 293.15 K			
0.0000	1133.4	1464.64	1.4875
0.1000	1116.6	1481.77	1.4839
0.2002	1099.0	1495.08	1.4799
0.2999	1080.7	1504.30	1.4755
0.4000	1061.8	1508.93	1.4709
0.5004	1042.2	1509.31	1.4659
0.5996	1022.6	1507.19	1.4609
0.6994	1002.5	1501.99	1.4554
0.7997	982.1	1494.75	1.4498
0.8997	961.7	1486.14	1.4441
1.0000	941.2	1475.92	1.4382
*T* = 303.15 K			
0.0000	1124.1	1432.57	1.4832
0.1000	1107.3	1448.23	1.4797
0.2002	1089.8	1459.96	1.4756
0.2999	1071.5	1468.53	1.4713
0.4000	1052.6	1472.40	1.4667
0.5004	1033.0	1471.71	1.4617
0.5996	1013.4	1469.32	1.4566
0.6994	993.3	1463.67	1.4512
0.7997	972.9	1455.68	1.4455
0.8997	952.5	1447.07	1.4398
1.0000	932.0	1436.08	1.4338
*T* = 313.15 K			
0.0000	1114.7	1400.34	1.4792
0.1000	1098.0	1415.01	1.4756
0.2002	1080.5	1425.45	1.4715
0.2999	1062.3	1433.03	1.4672
0.4000	1043.4	1435.56	1.4626
0.5004	1023.8	1434.65	1.4576
0.5996	1004.2	1431.72	1.4524
0.6994	984.1	1425.07	1.4470
0.7997	963.7	1417.05	1.4413
0.8997	943.3	1408.01	1.4354
1.0000	922.7	1396.63	1.4294
*T* = 323.15 K			
0.0000	1105.3	1369.16	1.4749
0.1000	1088.6	1382.09	1.4715
0.2002	1071.1	1391.43	1.4674
0.2999	1053.0	1397.95	1.4631
0.4000	1034.2	1400.11	1.4585
0.5004	1014.6	1398.13	1.4534
0.5996	995.0	1394.55	1.4483
0.6994	974.9	1387.43	1.4428
0.7997	954.5	1378.95	1.4369
0.8997	934.0	1369.41	1.4311
1.0000	913.5	1357.64	1.4251

a Standard uncertainties *u* are *u*(*T*) = 0.02 K and *u*(*p*) = 0.04 MPa, and the combined expanded uncertainties Uc in mole fraction, density, speed of sound, and refractive index are Uc(*x*) = ±0.0006, Uc(ρ) = 0.8 kg·m^–3^, Uc(*c*) = 2.81 m·s^–1^, and Uc(*n*_D_) = 0.0007, respectively (0.95 level of confidence).

**Figure 1:**
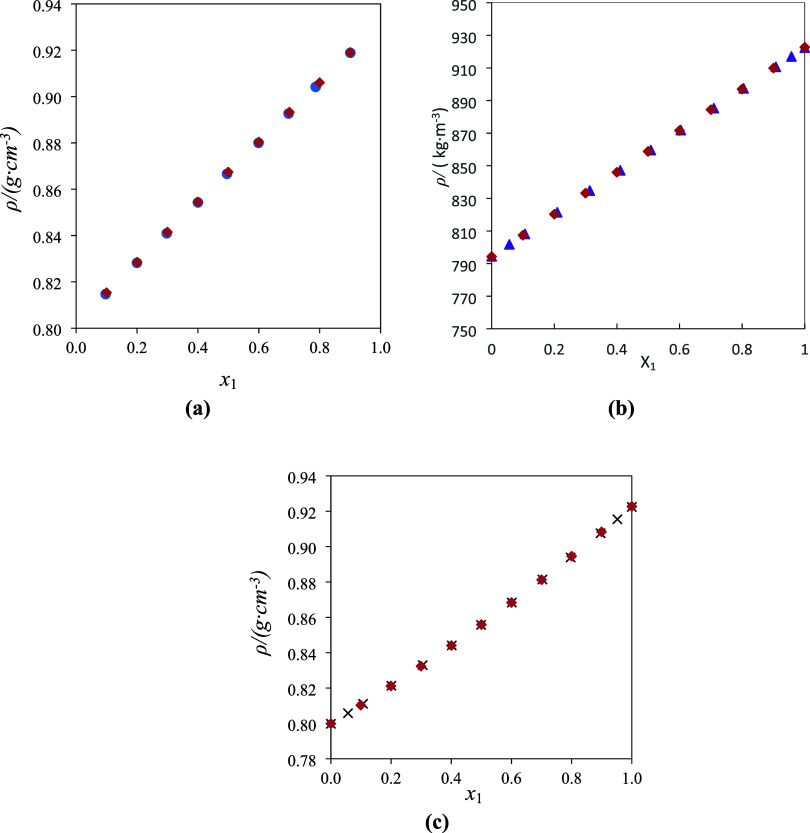
Plot of experimental density values with literature density values as a function of mole fraction (*x*_1_) of the binary systems: (a) DMA (1) + 1-butanol (2) at 303.15K, (b) DMA (1) + 1-pentanol (2) at 313.15 K (c), and DMA (1) + 1-butanol (2) at 313.15 K. Diamonds: experimental data from this work, circles: literature values reported by Pikkarainen,^25^ triangles: literature values reported by Mrad et al.,^26^ squares: and literature values reported by Mrad et al.^27^

It is observed that the ρ values increase with an increase in *x*_1_ for the DMA (1) + 1-butanol (2) and DMA + 1-pentanol (2) mixtures but decrease with an increase in temperature for the two binary mixtures. On the other hand, the ρ values for DMA (1) + FFL and DMA (1) + FA (2) decrease with an increase in *x*_1_, and also the obtained ρ values decrease with an increase in temperature. The experimental ρ values obtained in this work for DMA (1) + 1-butanol (2) and DMA (1) + 1-pentanol (2) binary systems were plotted with the literature data reported by Pikkarainen^25^ at *T* = 303.15 K, Mrad et al.^26^ at *T* = 313.15 K, and Mrad et al.^27^ at *T* = 313.15 K for comparison. As shown in Figure 1a–c, the data obtained in this work are consistent with those reported in the literature.

**Figure 2:**
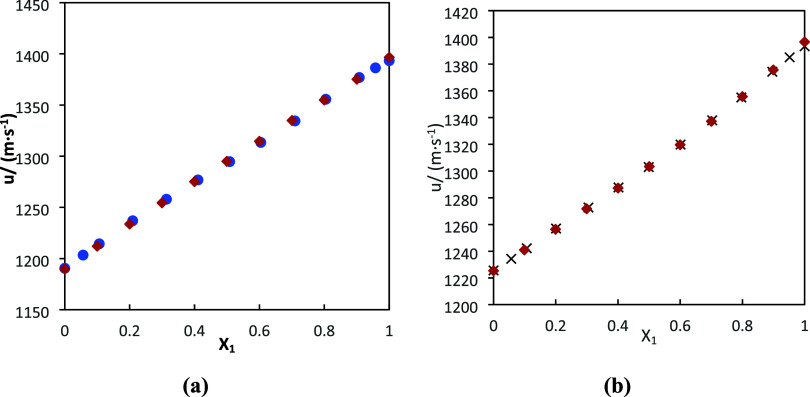
Plot of experimental speed of sound with literature values as a function of mole fraction (*x*_1_) for the (a) DMA (1) + 1-butanol (2) and (b) DMA (1) + 1-pentanol (2) binary systems at 313.15K. Diamonds: experimental data from this work, circles: literature values reported by Mrad et al.,^26^ and squares: literature values reported by Mrad et al.^27^

Figure 2a,b reports the comparison between the speed of sound experimental data for DMA (1) + 1-butanol (2) and DMA (1) + 1-pentanol (2) binary systems at 313.15 K and those reported by Mrad et al.^26,27^ As can be seen, the experimental values of *u* are in good agreement with the literature data.

**Figure 3:**
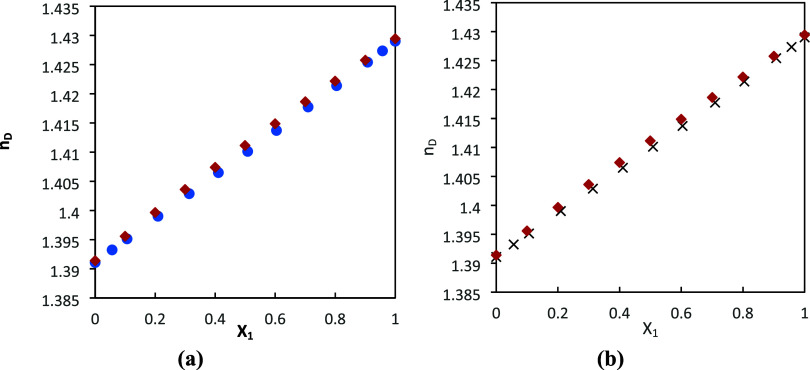
Plot of the experimental refractive index with literature values as a function of mole fraction (*x*_1_) for the (a) DMA (1) + 1-butanol (2) and (b) DMA (1) + 1-pentanol (2) binary systems at 313.15 K. Diamonds: experimental data from this work, circles: literature values reported by Mrad et al.,^26^ and squares: literature values reported by Mrad et al.^27^

The experimental refractive index values obtained in this work for DMA (1) + 1-butanol (2) and DMA (1) + 1-pentanol (2) binary systems were compared with values reported by Mrad et al.^26,27^ at *T* = 313.15 K. As shown in Figure 3a,b, our *n*_D_ values are slightly higher. This could be due to human manipulation during mixture preparation or measurement.

Further, the experimental values were used to compute the following thermodynamic, optical, and acoustic properties such as *κ*_s_*, L*_f_*, Ζ, R*_A_*, r,* and *R*. Table 3 presents the obtained values.

**Table 3:** Isentropic Compressibility (κ_s_), Intermolecular Free Length (*L*_f_), Specific Acoustic Impedance (*Z*), Relative Association (*R*_A_), Relaxation Strength (*r*), and Rao’s Molar Sound Function (*R*) for the Binary Systems *N*,*N*-Dimethylacetamide + 1-Butanol, 1-Pentanol, FFL, or FA at Different Temperatures and at Pressure *p* = 0.1 MPa^a^

***x*_1_**	**κ**_s_**/(TPa**^**–1**^**)**	*L*_f_**/(10**^**–12**^ **m)**	*Z***/(10**^**5**^ **kg m**^**–2**^ **s**^**–1**^**)**	***R*_A_**	***r***	*R***/(10**^**–3**^ **m(10/3) mol**^**–1**^ **s^–1/3^)**
DMA (1) + 1-butanol (2)						
*T* = 293.15 K						
0.0000	781.92	56.990	10.176	1.0000	0.3830	0.9878
0.0998	740.60	55.464	10.543	1.0103	0.3593	0.9949
0.2001	703.18	54.045	10.908	1.0206	0.3360	1.0018
0.2997	669.59	52.738	11.266	1.0310	0.3135	1.0085
0.3999	638.48	51.498	11.626	1.0413	0.2910	1.0151
0.4995	609.88	50.331	11.985	1.0516	0.2688	1.0216
0.5998	582.73	49.199	12.352	1.0619	0.2461	1.0281
0.7004	557.22	48.109	12.724	1.0721	0.2229	1.0346
0.7996	533.28	47.065	13.099	1.0820	0.1995	1.0410
0.9004	509.97	46.025	13.491	1.0920	0.1747	1.0474
1.0000	487.73	45.010	13.892	1.1018	0.1491	1.0538
*T* = 303.15 K						
0.0000	834.9	59.973	9.801	1.0000	0.4167	0.9880
0.0998	789.77	58.329	10.161	1.0099	0.3934	0.9954
0.2001	749.66	56.829	10.513	1.0201	0.3711	1.0025
0.2997	713.89	55.456	10.857	1.0304	0.3498	1.0093
0.3999	680.81	54.156	11.203	1.0407	0.3286	1.0159
0.4995	650.23	52.926	11.550	1.0510	0.3074	1.0224
0.5998	621.37	51.738	11.903	1.0612	0.2859	1.0289
0.7004	594.21	50.595	12.261	1.0714	0.2641	1.0354
0.7996	568.70	49.497	12.622	1.0813	0.2419	1.0418
0.9004	544.15	48.417	12.996	1.0914	0.2189	1.0482
1.0000	520.27	47.342	13.384	1.1011	0.1944	1.0546
*T* = 313.15 K						
0.0000	889.52	63.022	9.450	1.0000	0.4471	0.9888
0.0998	843.09	61.355	9.786	1.0101	0.4261	0.9960
0.2001	800.91	59.800	10.121	1.0203	0.4055	1.0031
0.2997	762.76	58.359	10.451	1.0306	0.3853	1.0099
0.3999	726.94	56.972	10.788	1.0408	0.3649	1.0167
0.4995	694.31	55.679	11.122	1.0510	0.3449	1.0232
0.5998	663.97	54.449	11.457	1.0614	0.3250	1.0295
0.7004	634.51	53.227	11.806	1.0715	0.3039	1.0361
0.7996	607.28	52.072	12.154	1.0814	0.2829	1.0425
0.9004	581.13	50.939	12.513	1.0915	0.2613	1.0489
1.0000	555.59	49.807	12.887	1.1012	0.2381	1.0554
*T* = 323.15 K						
0.0000	950.61	66.306	9.095	1.0000	0.4774	0.9894
0.0998	901.14	64.558	9.418	1.0101	0.4576	0.9967
0.2001	856.15	62.926	9.739	1.0203	0.4381	1.0038
0.2997	814.88	61.390	10.060	1.0304	0.4188	1.0107
0.3999	777.15	59.952	10.381	1.0407	0.3998	1.0174
0.4995	742.28	58.592	10.702	1.0510	0.3810	1.0239
0.5998	709.82	57.296	11.025	1.0614	0.3622	1.0303
0.7004	678.35	56.012	11.360	1.0715	0.3422	1.0369
0.7996	649.22	54.796	11.695	1.0814	0.3224	1.0433
0.9004	621.21	53.601	12.042	1.0915	0.3019	1.0496
1.0000	593.94	52.411	12.401	1.1012	0.2800	1.0561
DMA (1) + 1-pentanol (2)						
*T* = 293.15 K						
0.0000	735.21	55.262	10.527	1.0000	0.3479	1.1784
0.0991	706.98	54.190	10.806	1.0089	0.3307	1.1666
0.1993	680.23	53.155	11.092	1.0184	0.3138	1.1544
0.2994	654.55	52.142	11.385	1.0283	0.2966	1.1420
0.4004	629.71	51.143	11.689	1.0386	0.2790	1.1294
0.5007	605.32	50.143	12.008	1.0491	0.2606	1.1167
0.6000	581.77	49.158	12.337	1.0598	0.2417	1.1042
0.7002	558.06	48.146	12.690	1.0708	0.2211	1.0915
0.8000	534.55	47.121	13.064	1.0821	0.1990	1.0789
0.8994	510.95	46.069	13.466	1.0934	0.1748	1.0665
1.0000	487.73	45.010	13.892	1.1052	0.1491	1.0538
*T* = 303.15 K						
0.0000	781.89	58.037	10.162	1.0000	0.3812	1.1788
0.0991	752.18	56.924	10.428	1.0089	0.3651	1.1671
0.1993	723.95	55.846	10.701	1.0183	0.3492	1.1550
0.2994	696.81	54.789	10.982	1.0280	0.3330	1.1427
0.4004	670.58	53.748	11.274	1.0383	0.3165	1.1301
0.5007	644.85	52.706	11.578	1.0488	0.2993	1.1174
0.6000	619.87	51.675	11.894	1.0594	0.2814	1.1049
0.7002	594.74	50.617	12.233	1.0704	0.2620	1.0923
0.8000	569.82	49.545	12.592	1.0816	0.2412	1.0797
0.8994	544.85	48.448	12.976	1.0930	0.2185	1.0672
1.0000	520.27	47.342	13.384	1.1047	0.1944	1.0546
*T* = 313.15 K						
0.0000	831.22	60.922	9.810	1.0000	0.4125	1.1796
0.0991	801.28	59.814	10.057	1.0091	0.3984	1.1676
0.1993	771.42	58.689	10.318	1.0184	0.3834	1.1556
0.2994	742.74	57.588	10.586	1.0281	0.3682	1.1433
0.4004	714.95	56.500	10.866	1.0383	0.3527	1.1307
0.5007	687.62	55.410	11.158	1.0488	0.3364	1.1181
0.6000	661.25	54.337	11.460	1.0594	0.3197	1.1056
0.7002	634.59	53.230	11.784	1.0704	0.3015	1.0930
0.8000	608.18	52.111	12.128	1.0816	0.2820	1.0804
0.8994	581.70	50.964	12.496	1.0929	0.2607	1.0679
1.0000	555.59	49.807	12.887	1.1046	0.2381	1.0554
*T* = 323.15 K						
0.0000	887.70	64.075	9.447	1.0000	0.4446	1.1798
0.0991	854.59	62.868	9.691	1.0088	0.4305	1.1682
0.1993	822.93	61.693	9.941	1.0181	0.4164	1.1562
0.2994	792.54	60.543	10.199	1.0277	0.4021	1.1440
0.4004	763.07	59.407	10.466	1.0379	0.3875	1.1314
0.5007	734.08	58.267	10.746	1.0483	0.3723	1.1189
0.6000	706.09	57.145	11.035	1.0589	0.3566	1.1064
0.7002	677.81	55.990	11.346	1.0699	0.3395	1.0937
0.8000	649.78	54.819	11.675	1.0811	0.3212	1.0811
0.8994	621.66	53.620	12.027	1.0924	0.3013	1.0687
1.0000	593.94	52.411	12.401	1.1041	0.2800	1.0561
DMA (1) + FFL (2)						
*T* = 293.15 K						
0.0000	405.09	41.020	16.933	1.0000	0.1698	0.9379
0.1001	412.40	41.388	16.610	0.9790	0.1675	0.9491
0.2002	419.98	41.767	16.289	0.9584	0.1653	0.9603
0.3002	427.62	42.145	15.975	0.9382	0.1629	0.9717
0.3999	435.39	42.526	15.668	0.9184	0.1605	0.9831
0.4999	443.37	42.914	15.365	0.8990	0.1583	0.9946
0.6002	451.44	43.303	15.067	0.8798	0.1556	1.0064
0.6991	459.97	43.710	14.771	0.8612	0.1537	1.0181
0.7998	468.63	44.120	14.478	0.8426	0.1515	1.0300
0.9000	477.74	44.546	14.188	0.8246	0.1497	1.0419
1.0000	487.73	45.010	13.892	0.8070	0.1491	1.0538
*T* = 303.15 K						
0.0000	429.94	43.037	16.361	1.0000	0.2106	0.9387
0.1001	437.43	43.410	16.053	0.9789	0.2078	0.9499
0.2002	445.81	43.824	15.737	0.9584	0.2063	0.9611
0.3002	453.99	44.224	15.432	0.9382	0.2042	0.9725
0.3999	462.39	44.631	15.133	0.9184	0.2022	0.9840
0.4999	471.03	45.046	14.837	0.8990	0.2002	0.9955
0.6002	479.93	45.470	14.544	0.8798	0.1983	1.0072
0.6991	489.25	45.910	14.254	0.8612	0.1968	1.0189
0.7998	498.55	46.344	13.970	0.8426	0.1947	1.0310
0.9000	509.08	46.830	13.677	0.8247	0.1943	1.0427
1.0000	520.27	47.342	13.384	0.8071	0.1944	1.0546
*T* = 313.15 K						
0.0000	456.78	45.161	15.799	1.0000	0.2500	0.9394
0.1001	465.10	45.571	15.496	0.9790	0.2480	0.9506
0.2002	473.78	45.994	15.194	0.9584	0.2462	0.9619
0.3002	482.60	46.420	14.898	0.9382	0.2443	0.9733
0.3999	491.66	46.854	14.606	0.9184	0.2425	0.9848
0.4999	501.03	47.298	14.318	0.8990	0.2409	0.9962
0.6002	510.70	47.753	14.032	0.8798	0.2393	1.0080
0.6991	521.05	48.234	13.746	0.8613	0.2385	1.0197
0.7998	531.74	48.726	13.461	0.8427	0.2376	1.0316
0.9000	543.10	49.244	13.177	0.8247	0.2373	1.0435
1.0000	555.59	49.807	12.887	0.8071	0.2381	1.0554
*T* = 323.15 K						
0.0000	485.82	47.401	15.248	1.0000	0.2881	0.9401
0.1001	494.74	47.834	14.953	0.9790	0.2863	0.9513
0.2002	504.05	48.282	14.661	0.9584	0.2847	0.9627
0.3002	513.56	48.736	14.373	0.9382	0.2830	0.9741
0.3999	523.36	49.199	14.089	0.9184	0.2816	0.9855
0.4999	533.54	49.675	13.808	0.8990	0.2803	0.9970
0.6002	544.14	50.166	13.528	0.8798	0.2791	1.0088
0.6991	555.52	50.688	13.248	0.8613	0.2787	1.0204
0.7998	567.38	51.226	12.967	0.8428	0.2784	1.0323
0.9000	579.99	51.792	12.688	0.8248	0.2786	1.0442
1.0000	593.94	52.411	12.401	0.8072	0.2800	1.0561
DMA (1) + FA (2)						
*T* = 293.15 K						
0.0000	411.30	41.333	16.600	1.0000	0.1620	0.9829
0.1000	407.90	41.162	16.545	0.9813	0.1423	0.9904
0.2002	407.08	41.120	16.431	0.9630	0.1268	0.9978
0.2999	408.90	41.212	16.257	0.9451	0.1160	1.0052
0.4000	413.63	41.450	16.022	0.9276	0.1106	1.0122
0.5004	421.19	41.827	15.730	0.9104	0.1101	1.0192
0.5996	430.49	42.286	15.412	0.8937	0.1126	1.0261
0.6994	442.16	42.856	15.057	0.8771	0.1188	1.0329
0.7997	455.71	43.507	14.681	0.8607	0.1272	1.0398
0.8997	470.79	44.221	14.293	0.8444	0.1373	1.0468
1.0000	487.73	45.010	13.892	0.8283	0.1491	1.0538
*T* = 303.15 K						
0.0000	433.47	43.213	16.104	1.0000	0.1983	0.9838
0.1000	430.58	43.069	16.037	0.9815	0.1807	0.9911
0.2002	430.51	43.065	15.910	0.9633	0.1674	0.9983
0.2999	432.74	43.177	15.736	0.9454	0.1576	1.0057
0.4000	438.20	43.448	15.499	0.9279	0.1531	1.0128
0.5004	446.93	43.879	15.203	0.9108	0.1539	1.0197
0.5996	457.07	44.374	14.890	0.8939	0.1567	1.0266
0.6994	469.92	44.993	14.539	0.8773	0.1632	1.0335
0.7997	485.04	45.711	14.163	0.8609	0.1723	1.0404
0.8997	501.35	46.474	13.784	0.8445	0.1820	1.0476
1.0000	520.27	47.342	13.384	0.8284	0.1944	1.0546
*T* = 313.15 K						
0.0000	457.46	45.195	15.610	1.0000	0.2340	0.9845
0.1000	454.86	45.066	15.537	0.9816	0.2179	0.9918
0.2002	455.49	45.097	15.402	0.9635	0.2063	0.9989
0.2999	458.40	45.241	15.223	0.9456	0.1978	1.0062
0.4000	465.05	45.568	14.979	0.9283	0.1950	1.0131
0.5004	474.54	46.031	14.689	0.9111	0.1960	1.0201
0.5996	485.80	46.574	14.378	0.8942	0.1993	1.0271
0.6994	500.36	47.267	14.024	0.8777	0.2067	1.0340
0.7997	516.74	48.034	13.657	0.8611	0.2156	1.0410
0.8997	534.74	48.863	13.282	0.8447	0.2256	1.0482
1.0000	555.59	49.807	12.887	0.8285	0.2381	1.0554
*T* = 323.15 K						
0.0000	482.63	47.245	15.133	1.0000	0.2677	0.9855
0.1000	480.90	47.160	15.046	0.9818	0.2538	0.9925
0.2002	482.20	47.224	14.904	0.9639	0.2437	0.9995
0.2999	485.94	47.407	14.721	0.9461	0.2366	1.0067
0.4000	493.27	47.763	14.479	0.9287	0.2343	1.0137
0.5004	504.20	48.290	14.186	0.9116	0.2364	1.0206
0.5996	516.79	48.889	13.876	0.8947	0.2403	1.0276
0.6994	532.88	49.644	13.526	0.8781	0.2481	1.0345
0.7997	550.97	50.480	13.162	0.8615	0.2572	1.0416
0.8997	570.91	51.385	12.791	0.8450	0.2675	1.0489
1.0000	593.94	52.411	12.401	0.8288	0.2800	1.0561

a Standard uncertainties *u* are *u*(*T*) = 0.02 K and *u*(*p*) = 0.04 MPa, and the combined expanded uncertainties Uc in mole fraction, density, speed of sound, refractive index, isentropic compressibility, intermolecular free length, specific acoustic impedance, relative association, relaxation strength (*r*), and Rao’s molar sound function are Uc(*x*) = ± 0.0006, Uc(ρ) = 0.8 kg·m^–3^, Uc(*c*) = 2.81 m·s^–1^ and Uc(*n*_D_) = 0.0007, Uc(κ_s_) = 0.17 TPa^–1^, Uc(*L*_f_) = 0.006 × 10 ^–12^ m, Uc(*Z*) = 0.007 × 10^5^ kg m^–2^ s^–1^, Uc(*R*_A_) = 0.0009, Uc(*r*) = 0.0008, and Uc(*R*) = 0.0007 × 10^–3^ m(10/3) mol^–1^ s^–1/3^, respectively (0.95 level of confidence).

Isentropic compressibilities (κ_s_) were calculated using the Newton–Laplace equation:
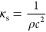
7

The intermolecular free length (*L*_f_) and specific acoustic impedance (*z*) were calculated using eqs 8 and 9, respectively:
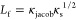
8
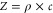
9where ρ and *c* are the density and sound velocity, respectively. *Κ*_jacob_ is the Jacobson temperature dependent; *Κ*_jacob_ = 93.875 + 0.375*T*)10^–8^.

The relative association (*R*_A_) was calculated using the following equation:
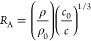
10where ρ, ρ_0_, and *c*, *c*_0_ are the densities and speeds of sound of the mixtures and solvents, respectively.

The relaxation strength (*r*) and Rao’s molar sound function (*R*) were determined using the following relations:
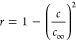
11
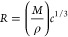
12

The value of the speed of sound at infinity *c*_∞_ is 1600 ms^–1^.

The excess and derived functions from ideality offer a better understanding of the nature and strength of the interaction between the constituent molecules of the binary mixtures.

### 4.1 Excess Molar Volumes

**Figure 4:**
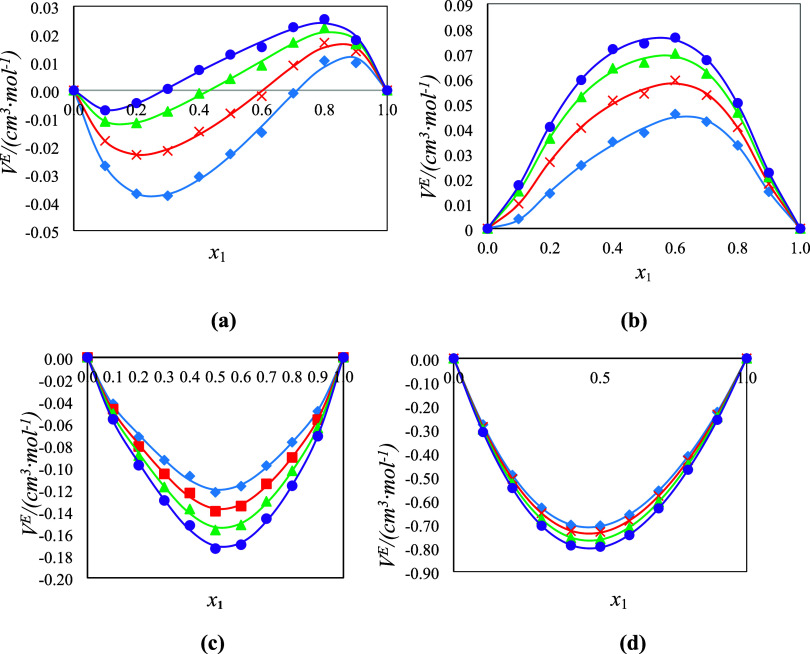
Plot of excess molar volumes (*V*_m_^E^) for the binary systems (a) DMA (1) + 1-butanol (2), (b) DMA (1) + 1-pentanol (2), (c) DMA (1) + FFL (2), and (d) DMA (1) + FA (2) as a function of composition expressed in mole fraction at *T* = 293.15 K (diamonds), 303.15 K (squares), 313.15 K (triangles), and 323.15 K (circles). The lines were generated using the Redlich–Kister polynomial curve fitting.

The excess molar volumes (*V*_m_^E^) for all the binary mixtures were derived from the experimental density data using eq 13 below:^21^
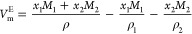
13where *V*_m_^E^ is excess molar volume; 1 denotes component 1 (DMA) and 2 denotes component 2 (1-butanol, 1-pentanol, FFL, or FA); and ρ, ρ_1_, and ρ_2_ are the densities of the binary mixtures DMA (1) and 1-butanol (2), 1-pentanol (2), FFL (2), or FA (2), respectively. *M*_1_ and *x*_1_ denote the molar mass and mole fraction of DMA (1), respectively, whereas *M*_2_ and *x*_2_ denote the molar mass and mole fraction of 1-butanol, 1-pentanol, FFL, or FA, respectively. The calculated *V*_m_^E^ of the four systems under study are shown in Table S1 on the Supporting Information. Furthermore, the plots of *V*_m_^E^ against *x*_*i*_ from 293.15 to 323.15 K of each binary system are presented in Figure 4a–d. The calculated *V*_m_^E^ show negative values for the DMA (1) + FFL (2) and DMA (1) + FA (2) systems over the whole range of compositions and become more negative with increasing temperature. The *V*_*m*_^E^ values for the DMA (1) + 1-pentanol (2) system are positive and increase with the increase in temperature, whereas a sinusoidal shape was obtained for the DMA (1) + 1-butanol (2) mixture with both negative and positive *V*_m_^E^ values in the 1-butanol and DMA rich mole fraction regions, respectively, as shown in Figure 4a–d.

The negative values may be due to the formation of strong cross hydrogen bonding interactions between dissimilar molecules (oxygen atom of the C=O group of DMA and the hydrogen atom of the hydroxyl group (−OH) of 1-butanol molecules). Furthermore, the positive values are due to the disruption of the self-associated 1-butanol molecules, which outweighs the cross-hydrogen bonding between the dissimilar molecules. The positive values for the DMA (1) + 1-pentanol (2) system can be attributed to the weakening of the dipole–dipole intermolecular interactions of DMA, disruption of the self-associated 1-pentanol molecules, and unfavorable packing of the unlike molecules. These results are in agreement with those obtained by Mrad et al.^27^ The negative values for the DMA (1) + FFL (2) and DMA (1) + FA (2) systems may be due to the strong intermolecular interactions arising from dipolar interactions, hydrogen bonding interactions, as well as charge transfer between the DMA (1) and the FFL (2) and FA (2) components.^28,29^

The obtained experimental values of densities, speeds of sound, and refractive indices for the pure compounds were compared with the literature^8,10,17,29−63^ values as shown in Table 4.

**Table 4:** Comparison of the Experimental Density (ρ), Speed of Sound (*c*), and Refractive Index (*n*_D_) of Pure Components with Literature Values at Different Temperatures and at Pressure *p* = 0.1 MPa^a^

**component**	*T***/(K)**	**ρ/(kg**·**m^–3^)**		*c/***(m**·**s**^**–1**^**)**		***n*_D_**	
		**exp.**	**lit.**	**exp.**	**lit.**	**exp.**	**lit.**
DMA	293.15	941.2	940.9^30^	1475.92	1475.30^10^	1.4382	1.4381^30^
			939.8^8^		1475.10^30^		
			939.8^31^		1472.00^8^		
	298.15	936.6	0936.4^32^	1456.02	1456.00^10^	1.4360	1.4359^30^
			936.3^33^		1455.70^30^		1.4359^32^
			936.3^27^		1458.00^36^		1.4358^27^
			936.2^34^		1453.68^37^		1.4357^35^
			936.3^35^		1462.10^38^		1.4356^39^
			935.3^31^				
			936.6^39^				
	303.15	932.0	931.7^30^	1436.08	1435.90^10^	1.4338	1.4337^10^
			931.5^8^		1435.70^30^		1.4338^30^
			930.8^31^		1432.00^8^		
	313.15	922.7	922.4^33^	1396.63	1393.35^37^	1.4294	1.4290^27^
			922.4^27^		1393.34^27^		1.4290^37^
			922.3^34^		1402.00^8^		
			923.4^8^				
			921.9^31^				
	323.15	913.5	913.2^40^	1357.64		1.4251	
1-pentanol	293.15	814.7	814.5^41^	1292.08	1293.27^43^	1.4098	1.4099^43^
			814.5^42^		1292.40^41^		
	298.15	811.1	811.0^44^	1275.38	1275.56^27^	1.4078	1.4078^43^
			811.0^27^		1276.84^43^		1.4079^48^
			810.2^45^		1274.90^47^		1.4080^49^
			810.6^46^				1.4077^44^
							1.4085^45^
	303.15	807.4	807.1^42^	1258.61		1.4059	1.4058^43^
			806.5^45^				1.4069^45^
			807.9^46^				
	313.15	799.9	799.0^45^	1226.37	1225.62^27^	1.4021	1.4017^43^
			799.8^27^		1228.25^43^		1.4039^45^
			799.6^42^				
			800.4^46^				
	323.15	792.3	792.2^50^	1192.41		1.3981	
			792.0^42^				
			792.7^46^				
FFL	293.15	1161.5	1160.1^51^	1457.83	1458.20^52^	1.5267	1.5264^52^
			1160.0^52^		1458.20^29^		1.5252^55^
			1160.0^53^		1458.52^51^		
			1160.0^54^		1459.06^53^		
			1159.3^29^				
	298.15	1156.2	1155.4^56^	1440.03	1440.17^52^	1.5242	1.5237^52^
			1155.0^53^		1440.21^53^		1.5231^17^
			1154.7^52^		1440.19^57^		
			1157.0^54^		1440.45^17^		
			1154.8^57^				
			1154.3^17^				
	303.15	1150.9	1151.0^55^	1421.59	1422.40^58^	1.5215	1.5212^52^
			1149.5^51^		1422.02^52^		1.5205^17^
			1149.2^58^		1422.05^53^		
			1149.3^52^		1422.50^29^		
			1149.0^53^		1422.73^17^		
			1149.0^17^				
			1148.7^29^				
	313.15	1140.2	1138.8^51^	1385.64	1385.97^51^	1.5163	1.5161^52^
			1138.4^58^		1386.00^58^		1.5152^17^
			1138.7^52^		1386.50^29^		
			1138.3^17^		1386.01^52^		
			1138.0^29^		1386.70^17^		
	323.15	1129.5	1128.1^51^	1349.97	1349.91^53^	1.5111	1.5111^50^
			1128.0^53^		1350.20^51^		
			1127.9^52^		1350.29^52^		
FA	293.15	1133.4	11349^51^	1464.64	1466.11^51^	1.4875	1.4874^55^
							1.4881^51^
	298.15	1128.8	1130.3^51^	1448.59	1449.80^51^	1.4853	1.4860^51^
			1126.9^57^		1447.25^57^		1.4845^57^
	303.15	1124.1	1125.6^51^	1432.57	1433.65^51^	1.4832	1.4839^51^
			1122.3^57^		1430.78^57^		1.4825^57^
	313.15	1114.7	1116.2^51^	1400.34	1401.71^51^	1.4792	1.4796^51^
			1112.9^57^		1394.33^57^		1.4781^57^
	323.15	1105.3	1106.8^51^	1369.16	1370.05^51^	1.4749	1.4753^51^
1-butanol	293.15	809.7	810.3^59^	1256.75	1258.13^60^	1.3992	1.3992^60^
			809.9^60^				
			809.6^58^				
	298.15	807.5	807.1^62^	1243.99	1241.32^60^	1.3972	1.3973^60^
			807.0^63^		1240.60^26^		1.3972^59^
			806.1^60^				1.3971^26^
			805.7^61^				1.3973^39^
			805.7^59^				
			806.1^26^				
			806^39^				
	303.15	802.1	803.7^63^	1222.01	1222.25^60^	1.3954	1.3953^60^
			802.3^60^				1.3953^59^
			801.9^59^				
			801.9^61^				
	313.15	794.3	796.7^63^	1189.68	1191.12^60^	1.3914	1.3913^60^
			794.5^60^		1190.52^26^		
			794.4^26^				1.3911^26^
	323.15	786.3	786.5^60^	1156.62	1158.00^60^	1.3874	1.3873^60^

a Standard uncertainties *u* are *u*(*T*) = 0.02 K and *u*(*p*) = 0.04 MPa, and the combined expanded uncertainties Uc in mole fraction, density, speed of sound, and refractive index are Uc(*x*) = ±0.0006, Uc(ρ) = 0.8 kg·m^–3^, Uc(*c*) = 2.81 m·s^–1^, and Uc(*n*_D_) = 0.0007, respectively (0.95 level of confidence).

### 4.2 Derived Properties

The calculated values such as κ_s_, *L*_f_, *Ζ*, *R*_A_, *r*, and *R* for the binary mixtures are plotted versus the mole fraction of DMA and are given in Figures S4–S9. An examination of data in Figure S7a–d shows increases of *R*_A_ for DMA + 1-butanol and DMA + 1-pentanol with increasing mole fraction of DMA and decreases for other systems DMA + FFL or FA. The increase in *R*_A_ indicates the existence of molecular interactions between molecules that are not similar. Higher values of the relative association indicate the strength of unlike interactions relative to like interactions. Figure S8a–d shows trends of variation in relaxation strength (*r*) versus mole fraction of DMA. It is evident that all binary systems exhibit nonlinear variation in *r* values, and it is found to decrease for all binary systems except {DMA + FA} system and increase with temperature at a constant DMA concentration.

It is possible to analyze molecular interactions in liquid mixtures using Rao’s molar sound function *R*.^10,64^ It is evident from Figure S9a–d that *R* increases linearly with DMA mole fraction for DMA + 1-butanol, DMA + FFL, and DMA + FA, whereas for DMA+ 1-pentanol, it is seams to decrease linearly. Several studies reported in the literature suggest that Rao’s molar sound function has a linear dependence on the molar fractions of the components if there is no association or if the degree of association is independent of the concentration.^10,64^ Moreover, there is no thermal impact observed on *R*_A_ and *R* values, which is similar to the literature.^65^

The excess isentropic compressibility (*k*_s_^E^) values were calculated for all the binary systems by using Benson and Kiyohara^66^ and Douheret et al.,^67^ and these values are given in Table S1.
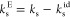
14Here, *k*_*s*_ is the isentropic compressibility, which was calculated from eq 7.

On the other hand, the ideal isentropic compressibilities were calculated from the equation below:
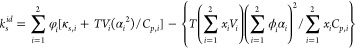
15where α_*i*_ is the thermal expansion coefficient and ϕ_*i*_ is the volume fraction that were calculated from eqs 16 and 17, respectively. *C*_*p,i*_ is the molar heat capacity that was obtained from the available literature.^68−71^
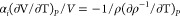
16
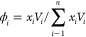
17

**Figure 5:**
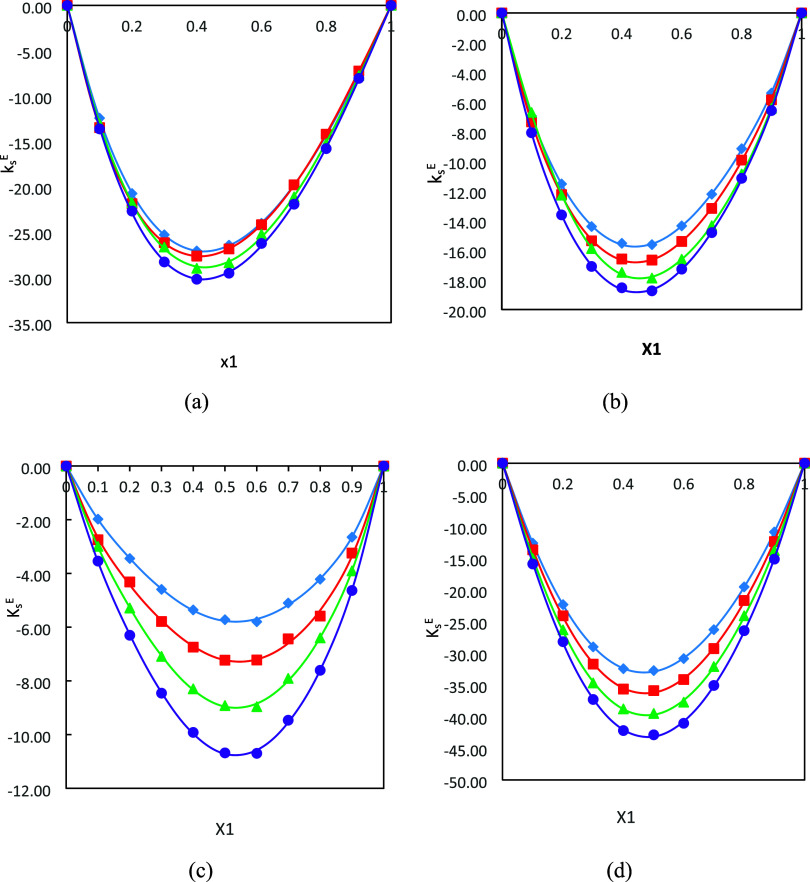
Plot of excess isentropic compressibilities (*k*_s_^E^) for the binary systems (a) DMA (1) + 1-butanol (2), (b) DMA (1) + 1-pentanol (2), (c) DMA (1) + FFL (2), and (d) DMA (1) + FA (2) as a function of composition expressed in mole fraction at *T* = 293.15 K (diamonds), 303.15 K (squares), 313.15 K (triangles), and 323.15 K (circles). The lines were generated using the Redlich–Kister polynomial curve fitting.

The plots of *k*_s_^E^ against mole fraction (*x*_*i*_) for each binary system under study are displayed in Figure 5a–d. In general, the *k*_s_^E^ values are negative for all four binary systems over the entire composition range, and the values decrease with the increase in temperature. This implies that these mixtures are less compressible than the corresponding pure components due to the strong interactions (dipole–dipole interactions, charge transfer complexes, and hydrogen bonding) between the unlike component molecules of the mixtures. In addition, the negative values are ascribed to the closer packing of the unlike molecules leading to the contraction in volume. In comparison, the *k*_s_^E^ values for the DMA (1) + 1-pentanol (2) and DMA (1) + FFL (2) systems are less negative, which may be attributed to the existence of weaker intermolecular interaction such as the van der Waals forces between the unlike molecules. The *k*_s_^E^ values for the DMA (1) + 1-butanol (2) and DMA (1) + FA (2) systems are found to be more negative in magnitude due to the existence of stronger forces of attraction.

**Figure 6:**
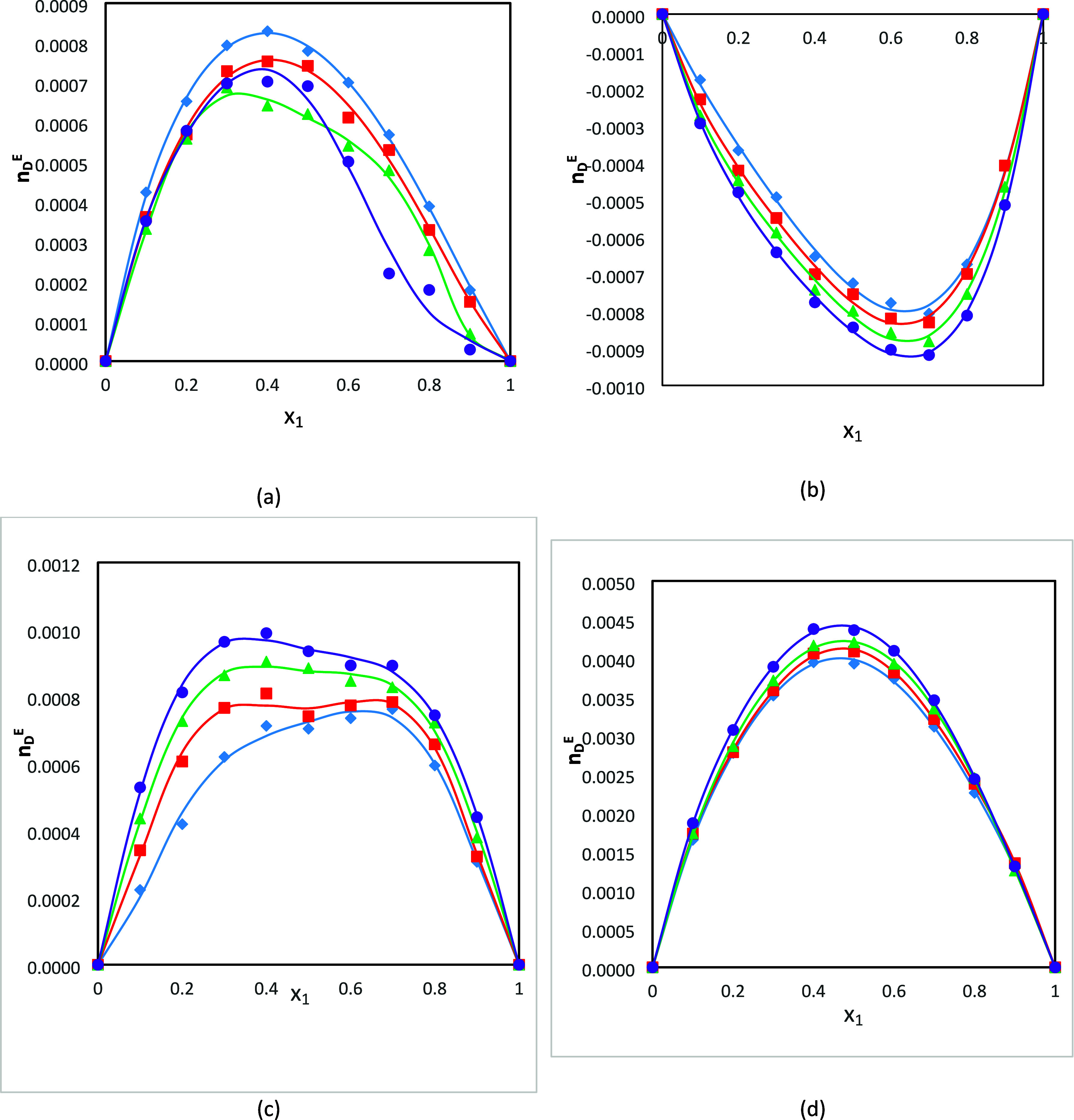
Plot of excess refractive indices (*n*_D_^E^) for the binary systems (a) DMA (1) + 1-butanol (2), (b) DMA (1) + 1-pentanol (2), (c) DMA (1) + FFL (2), and (d) DMA (1) + FA (2) as a function of composition expressed in mole fraction at *T* = 293.15 K (diamonds), 303.15 K (squares), 313.15 K (triangles), and 323.15 K (circles). The lines were generated using the Redlich–Kister polynomial curve fitting.

Excess refractive index (*n*_D_^E^) values were calculated from the following equation recommended by Resa et al.:^72^
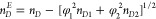
18where *n*_D_ is the refractive index of the mixture and *n*_D1_/*n*_D2_ and ϕ_1_/ϕ_2_ are the refractive index and volume fraction of component 1/2, respectively. The excess refractive index values of the binary mixtures at different temperatures are listed in Table S1, and the plot of excess refractive index (*n*_D_^E^) as function of composition expressed of mole fraction for the four binary systems is presented in Figure 6a–d. The positive curves are observed for the DMA (1) + 1-pentanol (2) binary system at *T* = 293.15 and 303.15 K and at the remaining temperatures (313.15 and 323.15 K) become sigmoidal curves. Also, it can be observed that the *n*_D_^E^ values for the DMA (1) + 1-butanol (2), DMA (1) + FA (2), and DMA (1) + FFL (2) binary systems are positive at all the studied temperatures.

### 4.3 Redlich–Kister Correlation

The Redlich–Kister polynomial equation^73^ was used to correlate the excess properties—excess molar volume (*V*_m_^E^), excess isentropic compressibility (*k*_s_^E^), and excess refractive index (*n*_D_^E^)—for the binary mixtures as a function of temperature according to the equation given below:
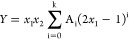
19

The fitting parameters *A*_*i*_ were determined by the least-squares regression method. The standard deviation σ is given by the following expression:
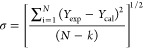
20where *Y*_exp_ is the experimental value, *Y*_cal_ is the calculated value from the Redlich–Kister equation, *N* is the number of experimental data points, and *k* is the number of adjustable parameters. The adjustable parameters and standard deviations are listed in Table 5. By comparing the experimental and calculated excess properties, it was clear that the Redlich–Kister model correlates the experimental data satisfactorily.

**Table 5:** Coefficients *A_i_* and Standard Deviations σ Obtained for the Binary Systems DMA + 1-Butanol, 1-Bentanol, FFL, FA for the Redlich–Kister Equation at Different Temperatures^a^

	*T* (K)	***A*_0_**	***A*_1_**	***A*_2_**	***A*_3_**	***A*_4_**	**σ**
*N*,*N*-Dimethylacetamide (1) + 1-butanol (2)							
*V*_m_^E^ (cm^3^·mol^–1^)	293.15	–0.093	0.194	0.012	0.116		0.002
	303.15	–0.035	0.157	0.030	0.117		0.001
	313.15	0.016	0.124	0.034	0.121		0.001
	323.15	0.049	0.106	0.031	0.119		0.001
*k*_s_^E^ (TPa^–1^)	293.15	–105.946	32.383	–10.608	2.813	3.409	0.0560
	303.15	–107.365	35.952	–15.577	11.482	4.323	0.0517
	313.15	–112.998	33.930	–7.255	2.950	3.570	0.2387
	323.15	–117.639	38.198	–12.192	–1.661	12.054	0.2104
*n*_D_^E^	293.15	0.0032	–0.0013	0.0003	–0.0005	0.0000	0.00001
	303.15	0.0029	–0.0012	–0.0001	–0.0004	–0.0001	0.00002
	313.15	0.0024	–0.0010	0.0022	–0.0012	–0.0041	0.00002
	323.15	0.0026	–0.0026	–0.0023	0.0007	0.0027	0.00005
*N*,*N*-Dimethylacetamide (1) + 1-pentanol (2)							
*V*_m_^E^ (cm^3^·mol^–1^)	293.15	0.161	0.118	0.050	–0.063	–0.224	0.001
	303.15	0.223	0.088	0.057	–0.050	–0.257	0.001
	313.15	0.273	0.064	0.063	–0.038	–0.282	0.001
	323.15	0.302	0.054	0.055	–0.027	–0.287	0.001
*k*_s_^E^ (TPa^–1^)	293.15	–62.328	13.231	–3.918	–2.627	–9.245	0.0751
	303.15	–66.592	13.345	–6.037	–3.445	–6.888	0.0736
	313.15	–71.184	11.166	–4.713	–12.181	2.656	0.1119
	323.15	–74.671	14.205	–5.518	–4.360	–8.034	0.0916
*n*_D_^E^	293.15	0.0015	–0.0014	–0.0007	0.0002		0.0000
	303.15	0.0012	–0.0013	–0.0009	0.0006		0.0000
	313.15	0.0010	–0.0014	–0.0014	0.0004		0.0000
	323.15	0.0007	–0.0012	–0.0017	–0.0003		0.0000
*N*,*N*-Dimethylacetamide (1) + FFL (2)							
*V*_m_^E^ (cm^3^·mol^–1^)	293.15	–0.480	–0.044	0.197	0.018	–0.384	0.003
	303.15	–0.549	–0.072	0.193	0.029	–0.380	0.003
	313.15	–0.617	–0.094	0.204	0.027	–0.387	0.003
	323.15	–0.684	–0.123	0.210	0.046	–0.405	0.003
*k*_s_^E^ (TPa^–1^)	293.15	–23.075	–3.216	0.457	–2.100	–7.154	0.0695
	303.15	–28.906	–5.159	–1.550	0.674	–8.732	0.1639
	313.15	–35.838	–5.272	1.544	–1.408	–8.556	0.0795
	323.15	–42.876	–6.319	2.288	–1.601	–9.851	0.0820
*n*_D_^E^	293.15	0.0029	0.0008	0.0026	0.0000	–0.0041	0.00003
	303.15	0.0031	0.0001	0.0048	–0.0001	–0.0061	0.00003
	313.15	0.0035	–0.0002	0.0041	0.0000	–0.0037	0.00002
	323.15	0.0038	–0.0005	0.0042	0.0001	–0.0027	0.00002
*N*,*N*-Dimethylacetamide (1) + FA (2)							
*V*_m_^E^ (cm^3^·mol^–1^)	293.15	–2.836	0.457	0.026	–0.134		0.002
	303.15	–2.942	0.461	0.028	–0.145		0.002
	313.15	–3.061	0.461	0.031	–0.144		0.002
	323.15	–3.194	0.465	0.040	–0.164		0.002
*k*_s_^E^ (TPa^–1^)	293.15	–131.636	17.496	2.032	–7.962		0.0965
	303.15	–144.877	16.581	2.059	–11.321		0.2311
	313.15	–158.862	15.445	0.679	–9.905		0.2517
	323.15	–172.680	14.650	2.872	–14.381		0.2489
*n*_D_^E^	293.15	0.0159	–0.0024	–0.0015	–0.0003	0.0031	0.00004
	303.15	0.0164	–0.0022	–0.0029	–0.0005	0.0059	0.00003
	313.15	0.0168	–0.0018	–0.0003	–0.0019	–0.0004	0.00004
	323.15	0.0176	–0.0024	–0.0017	–0.0023	0.0023	0.000037

a Standard uncertainties *u* are *u*(T) = 0.02 K and *u*(*p*) = 0.04 MPa, and the combined expanded uncertainties Uc in mole fraction, density, speed of sound, refractive index, excess molar volume, deviation in isentropic compressibility and deviation in refractive intermolecular free length, specific acoustic impedance, relative association, relaxation strength (*r*), and Rao’s molar sound function are Uc(*x*) = ± 0.0006, Uc(ρ) = 0.8 kg·m^–3^, Uc(*c*) = 2.81 m·s^–1^ and Uc(*n*^D^) = 0.0007, Uc(*V*_m_^E^) = 0.003 cm^3^·mol^–1^, Uc(*k*_s_^E^) = 0.17 TPa^–1^, Uc(*n*_D_^E^) = 0.0008, Uc(*L*_f_) = 0.006 × 10 ^–12^ m, Uc(*Z*) = 0.007 × 10^5^ kg m^–2^ s^–1^, Uc(*R*_A_) = 0.0009, Uc(*r*) = 0.0008, and Uc(*R*) = 0.0007 × 10^–3^ m(10/3) mol^–1^ s^–1/3^, respectively (0.95 level of confidence).

### 4.4 Modeling of Density, Speed of Sound, and Refractive Index

The pure-fluid parameters required in PC-SAFT were optimized in this article using the correlations of vapor pressure published by DIPPR and the experimental density data obtained in this work. The objective function (OF) is given by eq 21:
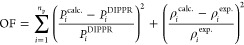
21where *n*_p_ is the number of experimental points, *P* is the vapor pressure, ρ is the density, calc*.* is calculated using PC-SAFT EoS, and exp. is experimental. Also, the absolute average deviation is calculated as eq 22:
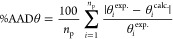
22with θ: density, speed of sound, or refractive index.

Table 6 shows the pure-fluid parameters of PC-SAFT and the statistical deviation in liquid density.

**Table 6:** Parameters for the Pure Fluids Required in the PC-SAFT EoS and the Absolute Average Deviation for the Liquid Density^a^

fluid	*m*	σ/(Å)	*ε*/*k*_B_/(K)	ε^AB^/*k*_B_/(K)	κ^AB^	%AADρ
DMA	2.9500	3.5346	328.106			0.09
1-butanol	2.9000	3.5250	259.004	2472.38	0.006217	0.01
1-pentanol	2.8000	3.8007	291.271	2564.84	0.002074	0.05
FFL	3.0000	3.3828	324.554			0.02
FA	2.8500	3.4702	238.900	3029.13	0.048661	0.01
overall						0.04

a The temperature range was 293.15 to 323.15 K.

According to this table, an overall deviation of 0.04% was obtained, and therefore, PC-SAFT correctly adjusts the liquid density of pure liquids. Subsequently, once the parameters of the pure fluids have been obtained, it is possible to perform calculations for the mixtures. The deviations obtained in density, speed of sound, and refractive index are published in Table 7.

**Table 7:** Statistical Deviation Calculated Using the Temperature Range 293.15–323.15 K for Density, Speed of Sound, and Refractive Index for Mixtures

**mixture**	%**AADρ**	%**AAD*c***		%**AAD*n*_D_**			
		**SCFT**	**NR**	**LL**	**GD**	**LP**	**EK**
DMA (1) + 1-butanol (2)	0.07	0.23	0.23	0.06	0.05	0.04	0.06
DMA (1) + 1-pentanol (2)	0.14	0.26	0.08	0.08	0.07	0.05	0.07
DMA (1) + FFL (2)	0.10	0.12	0.12	0.08	0.04	0.01	0.05
DMA (1) + FA (2)	0.54	1.72	1.87	0.20	0.19	0.18	0.19
overall	0.21	0.58	0.58	0.11	0.09	0.07	0.09

From this table, it is observed that PC-SAFT correctly predicts the density for all liquid mixtures (overall deviation of 0.21%). In the case of the speed of sound, both models (SCFT and NR) provide approximately the same theoretical results, except for the mixtures of DMA with 1-pentanol and FA. With both models, deviations close to 2% were obtained for the DMA + FA mixture; this low value does not guarantee a good qualitative agreement (this will be discussed later with the help of a figure). On the other hand, all mixing rules (LL, GD, LP, and EK) are capable of correctly modeling the refractive index of the mixtures; the best model was LP (overall deviation of 0.07%).

**Figure 7:**
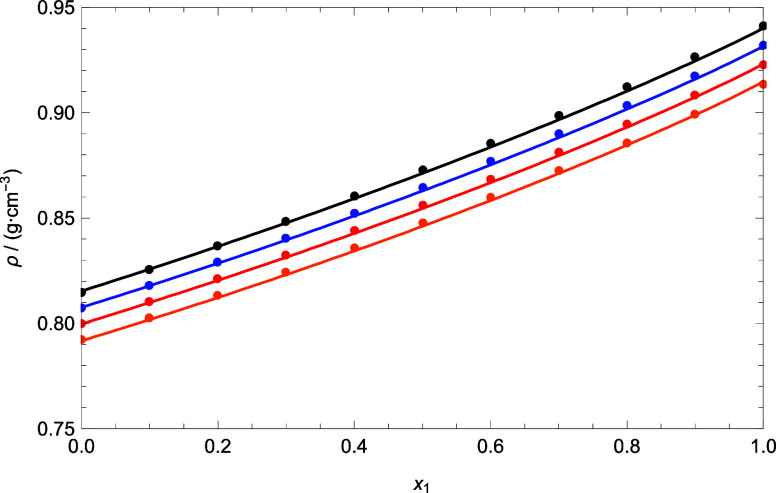
Comparison between experimental data and theoretical data of density for the DMA (1) + 1-pentanol (2) at different temperatures using PC-SAFT EoS. Colors: (black) 293.15 K, (blue) 303.15 K, (red) 313.15 K, and (orange) 323.15 K.

Figure 7 shows the variation of liquid density with the mole fraction of DMA for the mixture DMA (1) + 1-pentanol (2) at different temperatures. According to this figure, there is good agreement between the theoretical and experimental data at different temperatures. The model correctly represents the variation with the temperature. Good results were obtained for this mixture and for the other mixtures (which are supported by the results in Table 7). On the other hand, SCFT and NR allow good theoretical results for DMA + 1-butanol and DMA + 1-pentanol but not so for DMA + FFL and DMA + FA, whereas for the refractive index, all the rules for the refractive index correctly model the experimental data at different temperatures. It is important to mention that the equation of state is a good choice of EoS selected; however, in the future, it would be interesting to investigate mixing rules for the speed of sound with fitted parameters to improve the representation of experimental data. Also, the excess molar volume data (*V*_m_^E^) for all the studied binary systems were compared with the PC-SAFT EoS method at different temperatures in Figure S10.

**Figure 8:**
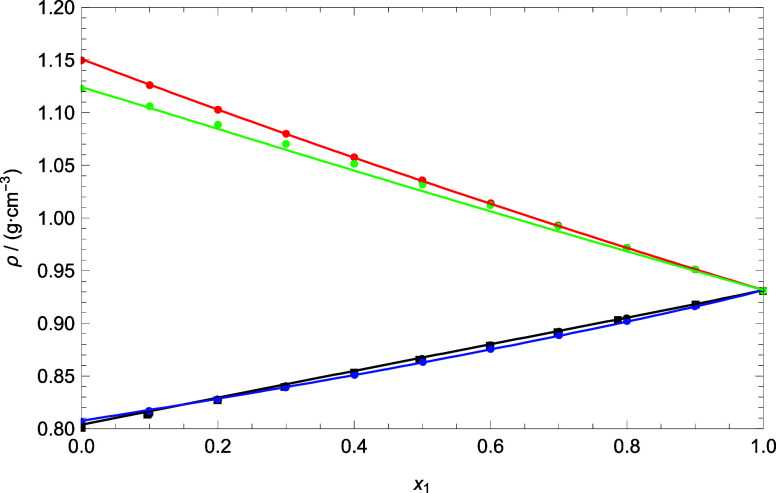
Comparison between experimental data and theoretical data of density for all of the binary mixtures at 303.15 K. Circles represent the experimental data obtained in this work. Squares represent the experimental data of the literature^71^ for DMA (1) + 1-butanol (2). Lines were generated using PC-SAFT EoS. Colors: (black) DMA (1) + 1-butanol (2), (blue) DMA (1) + 1-pentanol (2), (red) DMA (1) + FFL (2), and (green) DMA (1) + FA (2).

**Figure 9:**
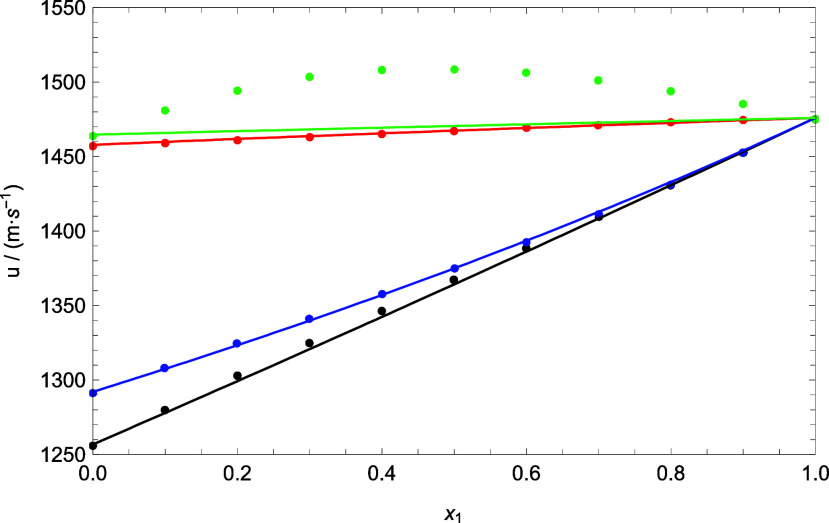
Comparison between experimental data and theoretical data of speed of sound for all of the binary mixtures at 293.15 K. Circles represent the experimental data. Lines were generated using PC-SAFT EoS + NR. Colors: (black) DMA (1) + 1-butanol (2), (blue) DMA (1) + 1-pentanol (2), (red) DMA (1) + FFL (2), and (green) DMA (1) + FA (2).

**Figure 10:**
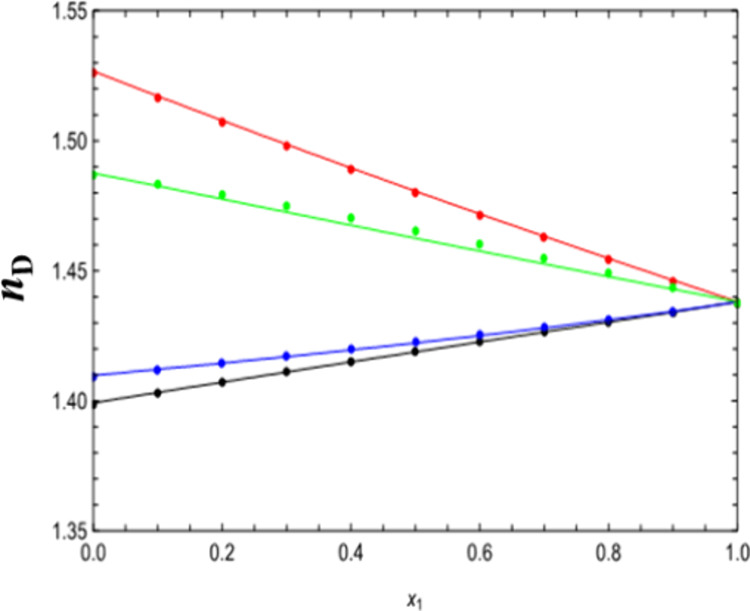
Comparison between experimental data and theoretical data of the refractive index for all the binary mixtures at 293.15 K. Circles represent the experimental data. Lines were generated using the PC-SAFT EoS + LP mixing rule. Colors: (black) DMA (1) + 1-butanol (2), (blue) DMA (1) + 1-pentanol (2), (red) DMA (1) + FFL (2), and (green) DMA (1) + FA (2).

Figure 8 shows a comparison of the density of the mixtures at 303.15 K, whereas Figures 9 and 10 show a comparison of the speed of sound and refractive index at 293.15 K, respectively. According to these figures, good qualitative and quantitative agreement between the experimental density and the values predicted by PC-SAFT EoS was obtained. Furthermore, the trends of increasing and decreasing density with mole fraction of DMA are well captured by PC-SAFT. The attractive forces between molecules are well modeled with PC-SAFT. Also, cross-association allows hydrogen bond forces to be correctly modeled. On the other hand, doing a review of the literature, we have found only the work of Pikkarainen^25^ that publishes experimental density data for the mixture DMA + 1-butanol at 303.15 K. We have calculated the deviation in density of our model, and a value of 0.08% was obtained. Therefore, our model correctly represents the literature data.

Figure 9 illustrates the comparison of the speed of sound. The good agreement of PC-SAFT + NR is observed for all mixtures, except for the DMA + FA mixture, where it is clearly observed that these results do not make physical sense (despite a deviation of 1.87%; low deviation due to the small range of speed of sound values for this mixture) because the prediction does not correctly capture the quadratic trend of the experimental data. This can be explained by the fact that, perhaps, this mixture requires some fitted parameters to experimental values for the speed of sound or that the null parameter *k*_*ij*_ does not always guarantee good sound speed prediction results.

On the other hand, Figure 10 illustrates the comparison of the refractive index, where a good qualitative and quantitative agreement is observed for all mixtures using the PC-SAFT EoS + Laplace mixing rule. Finally, it is very important to highlight that a completely predictive approach has been used in modeling the density, speed of sound, and refractive index for the binary mixtures at different temperatures.

## 5 Conclusions

In the present work, four novel binary systems—DMA (1) + 1-butanol (2), + 1-pentanol (2), + FFL (2), and + FA (2)—were successfully prepared and studied to understand the molecular interactions that occur between the components of the investigated systems. The experimental data values were used to calculate the excess properties, which were then correlated using a Redlich–Kister polynomial equation. The excess molar volumes were negative for the DMA (1) + FFL (2) and DMA (1) + FA (2) systems and positive for the DMA (1) + 1-pentanol system. On the other hand, the excess molar volume for the DMA (1) + 1-butanol system was sigmoidal. The *k*_s_^E^ values are negative for all the four binary systems over the entire composition range, and the values decrease with the increase in temperature. In addition, the *n*_D_^E^ values for DMA (1) + 1-butanol (2), DMA (1) + FFL (2), and DMA (1) + FA (2) systems are negative, whereas those for the DMA (1) + 1-pentanol (2) system are positive at 2913.15 and 303.15 K, and the remaining temperatures become sigmoidal curves. Appreciably, the significant changes in the excess properties under study have confirmed the existence of the molecular interactions between the DMA and the 1-butanol, 1-pentanol, FFL, or FA systems. PC-SAFT correctly modeled as a predictive approach the density of all binary mixtures (overall deviation of 0.21%). SCFT and NR were able to correctly model the speed of sound of all mixtures, except for DMA + FA. Finally, the four mixing rules used allowed us to correctly predict the trend of the experimental data; LP was the best model with an overall deviation of 0.07%.

## Supporting Information Available

Thermophysical/thermodynamic properties of binary systems (a) DMA + 1-butanol, (b) DMA+ 1-pentanol, (c) DMA+ FFL, and (d) DMA+ FA at 293.15, 303.15, 313.15, and 323.15 K and at pressure *p* = 0.1 MPa (PDF)
