# Supplementary material for: Density, Speed of Sound, Refractive Index, and the Derived Properties of Binary Mixtures of N,N-Dimethylacetamide with 1-Butanol, 1-Pentanol, Furfural, or Furfuryl Alcohol at Different Temperatures
Source: J Chem Eng Data. 2024 Nov 14;70(1):67–86. doi: 10.1021/acs.jced.4c00275 (PMC11726579; doi:10.1021/acs.jced.4c00275)
Supplement: Supplementary file 1 — je4c00275_si_001.pdf [file je4c00275_si_001.pdf]

Supporting Information

Density, speed of sound, refractive index, and their derived properties of binary mixtures of N, N-dimethylacetamide with 1-butanol, 1-pentanol, furfural or furfuryl alcohol at different temperatures

Joan Chepkoech Kilele[1,2], Amal Ayad[1], Joseph Saab[3], Amina Negadi[1], Ariel Hernández[4], Indra Bahadur[5,*], Vibha Kumar[6], Mostafizur Rahaman[7], Latifa Negadi[1,8,*]

[1]LATA2M, Laboratoire de Thermodynamique Appliquée et Modélisation Moléculaire, University of Tlemcen, Post Office Box 119, Tlemcen 13000, Algeria

[2]Department of Chemistry, Durban University of Technology, Durban 4001, South Africa

[3]Department of Chemistry Biochemistry, Holy Spirit University of Kaslik, Faculty of Arts and Sciences USEK, ThEA Group Thermodynamic, Phase Equilibria, and Analysis, Post Office box 446, Jounieh, Lebanon

[4]Departamento de Ingeniería Industrial, Facultad de Ingeniería, Universidad Católica de la Santísima Concepción, Alonso de Ribera 2850, Concepción, Chile

[5]Department of Chemistry, North-West University (Mafikeng Campus), Private Bag X2046, Mmabatho 2735, South Africa

[6]Department of Chemistry, Pt. L. M. S. Campus, Rishikesh, SDS University, Badshahithaul 249201, India

[7]Department of Chemistry, College of Science, King Saud University, P.O. Box 2455, Riyadh 11451, Saudi Arabia

[8]Thermodynamics Research Unit, School of Engineering, University of KwaZulu-Natal, Howard College Campus, King George V Avenue, Durban 4041, South Africa

***Corresponding authors:** latifa.negadi@univ-tlemcen.dz; latifanegadi@yahoo.fr; bahadur.indra@nwu.ac.za.

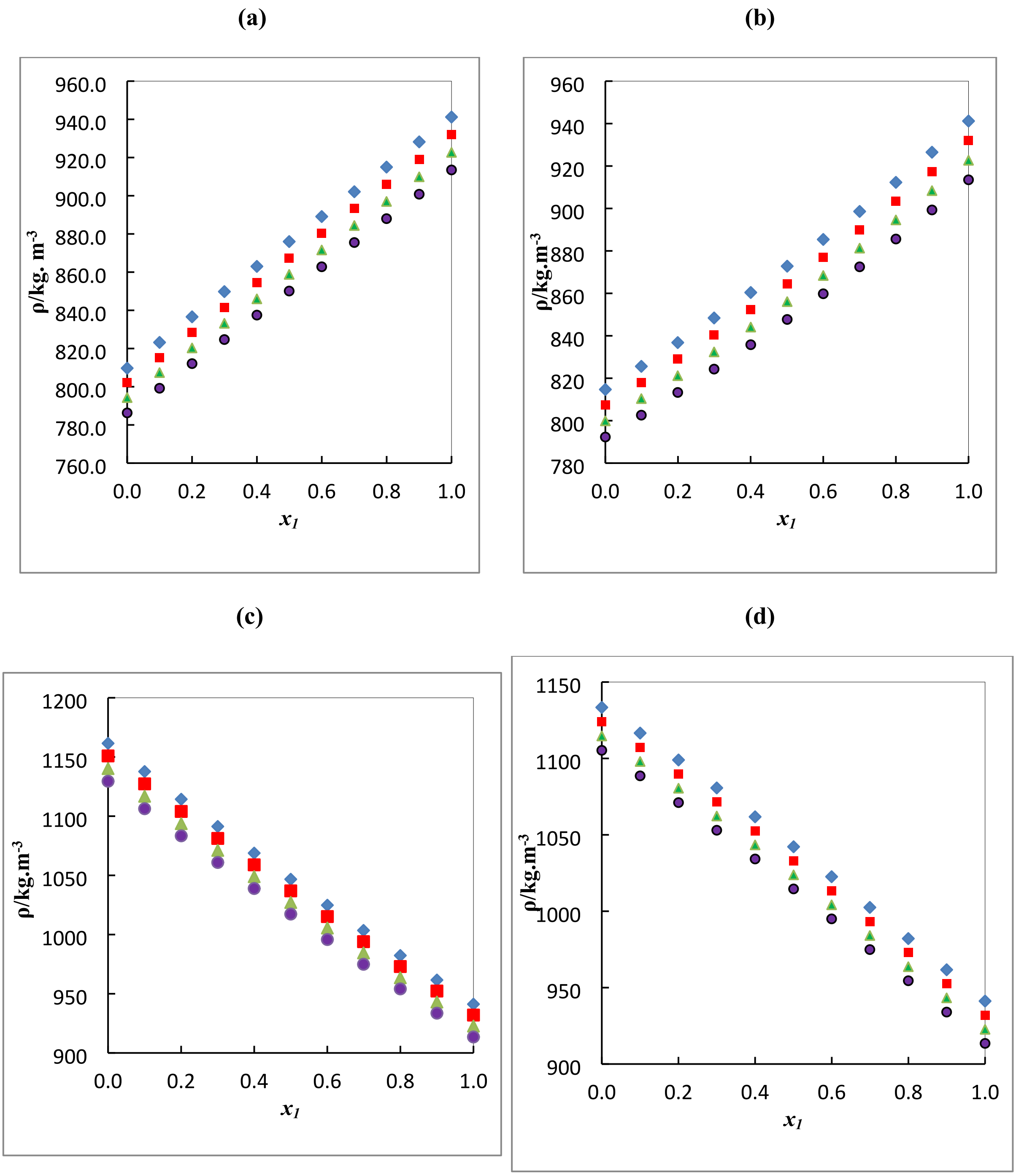

Fig. S1. Plot of density (ρ) for the binary mixtures: (a) {DMA + 1-butanol}. (b) {DMA+ 1-pentanol}. (c) {DMA+ FFL} and (d) {DMA+ FA} as function of the mole fraction of DMA at 293.15 K (◆), 303.15 K (■), 313.15 K (▲), 323.15 K (●).

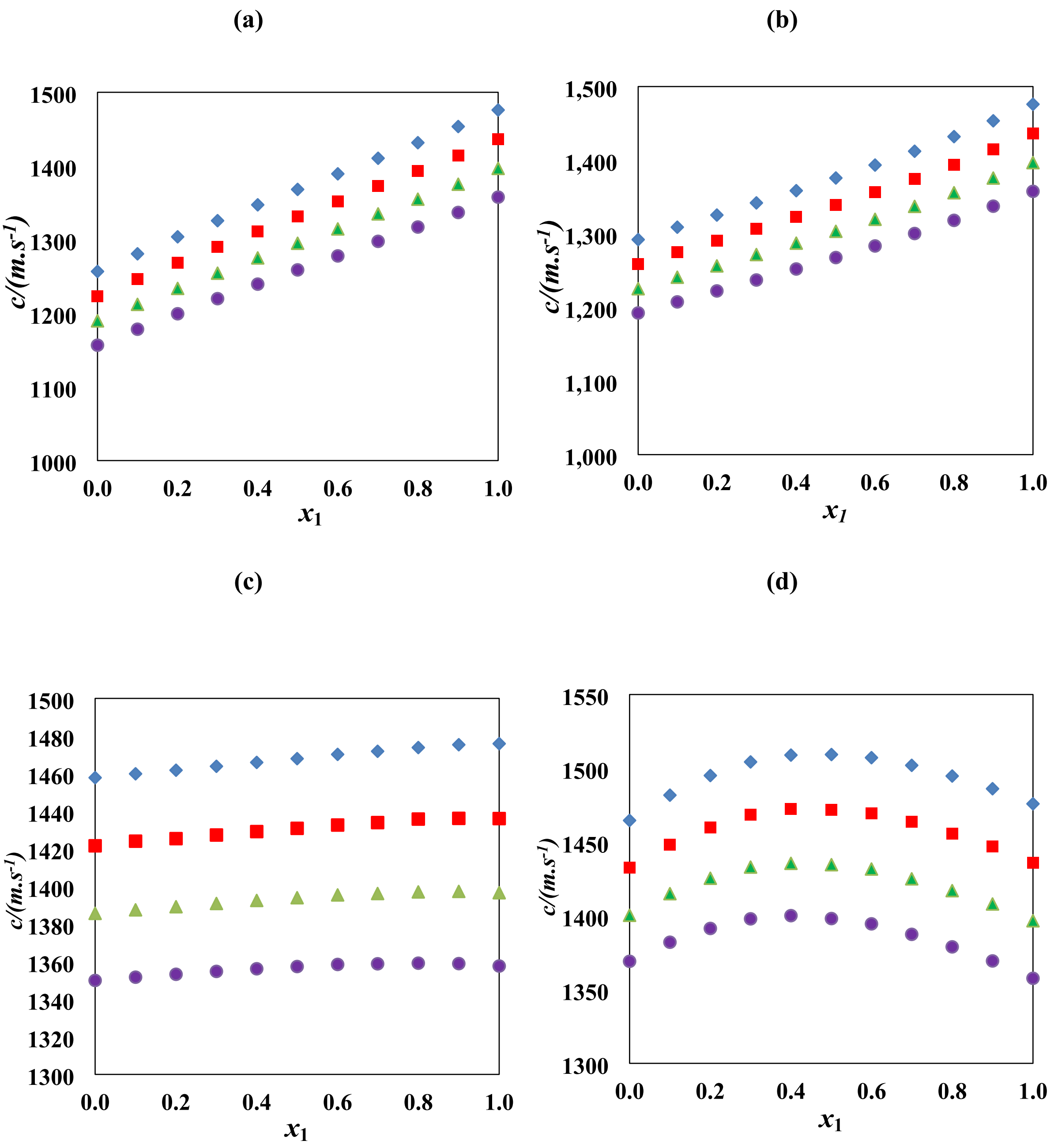

Fig. S2. Plot of speed of sound (c) for the binary mixtures: (a) {DMA + 1-butanol}, (b) {DMA+ 1-pentanol}, (c) {DMA+ FFL} and (d) {DMA+ FA} as function of the mole fraction of DMA at 293.15 K (◆), 303.15 K (■), 313.15 K (▲), 323.15 K (●).

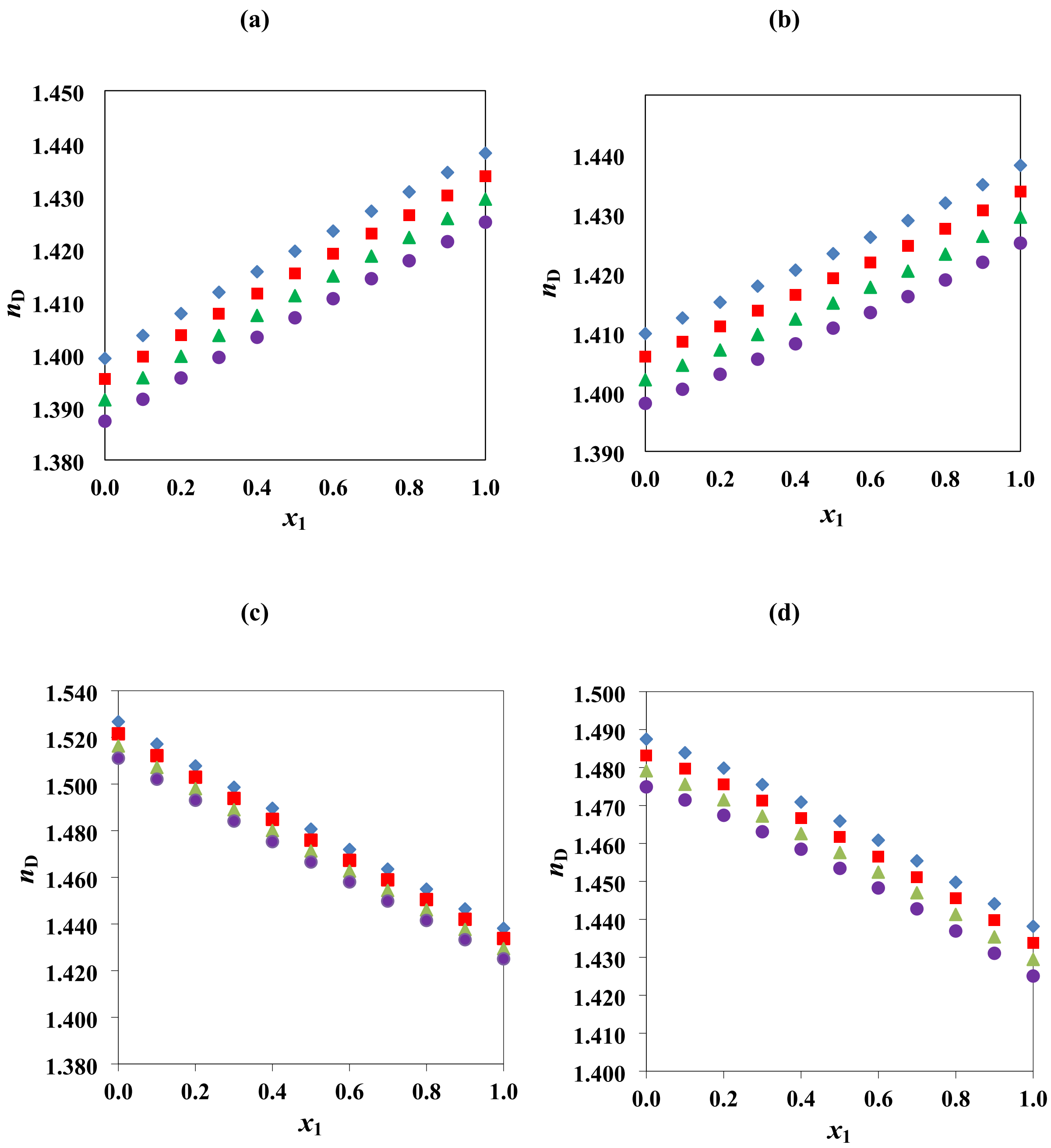

Fig. S3. Plot of refractive index (n_D) for the binary mixtures: (a) {DMA + 1-butanol}, (b) {DMA+ 1-pentanol}, (c) {DMA+ FFL} and (d) {DMA+ FA} as function of the mole fraction of DMA at 293.15 K (◆), 303.15 K (■), 313.15 K (▲), 323.15 K (●).

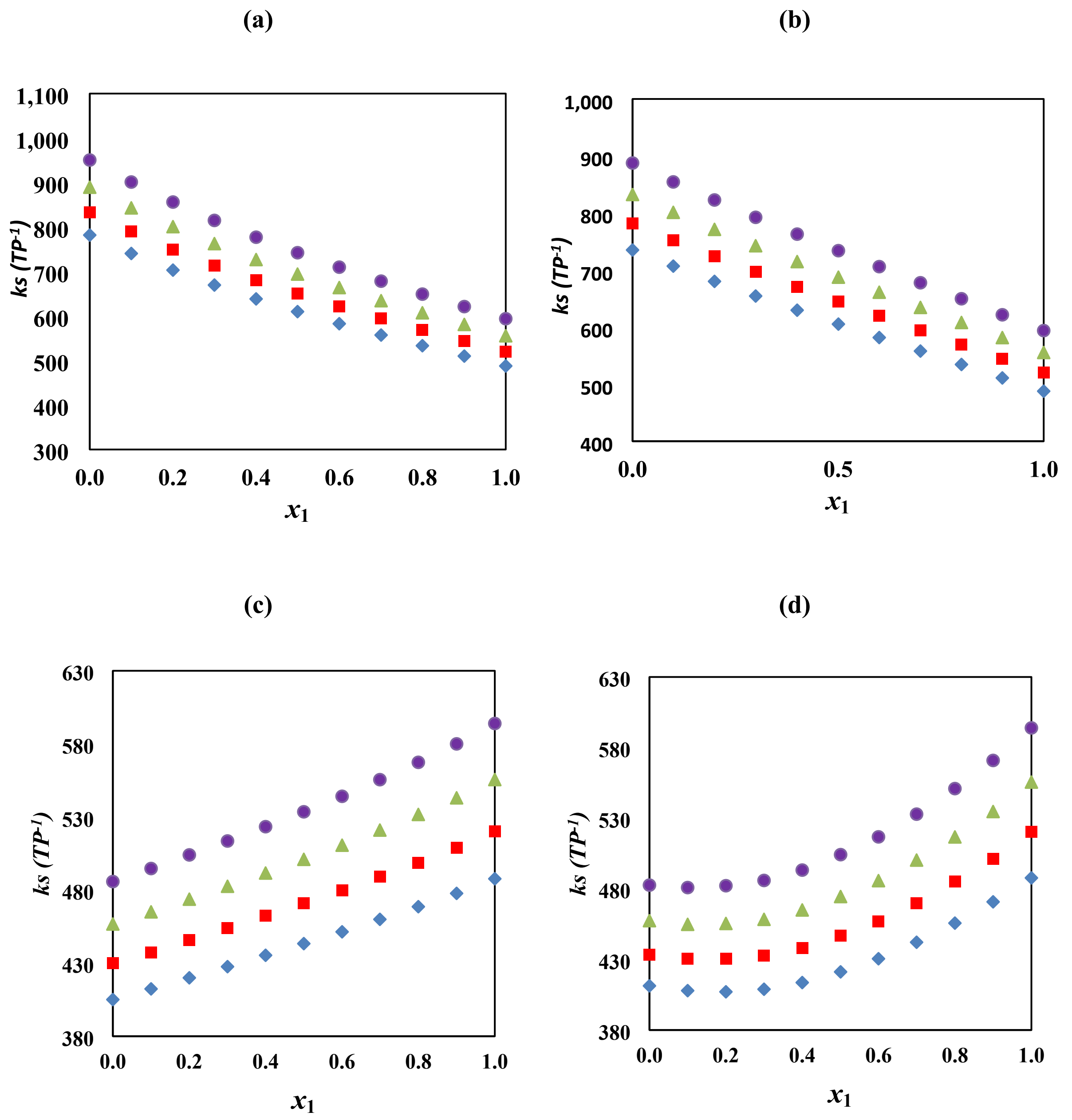

Fig. S4. Plot of isentropic compressibility (κ_s) for the binary mixtures: (a) {DMA + 1-butanol}, (b) {DMA+ 1-pentanol}, (c) {DMA+ FFL} and (d) {DMA+ FA} as function of the mole fraction of DMA at 293.15 K (◆), 303.15 K (■), 313.15 K (▲), 323.15 K (●).

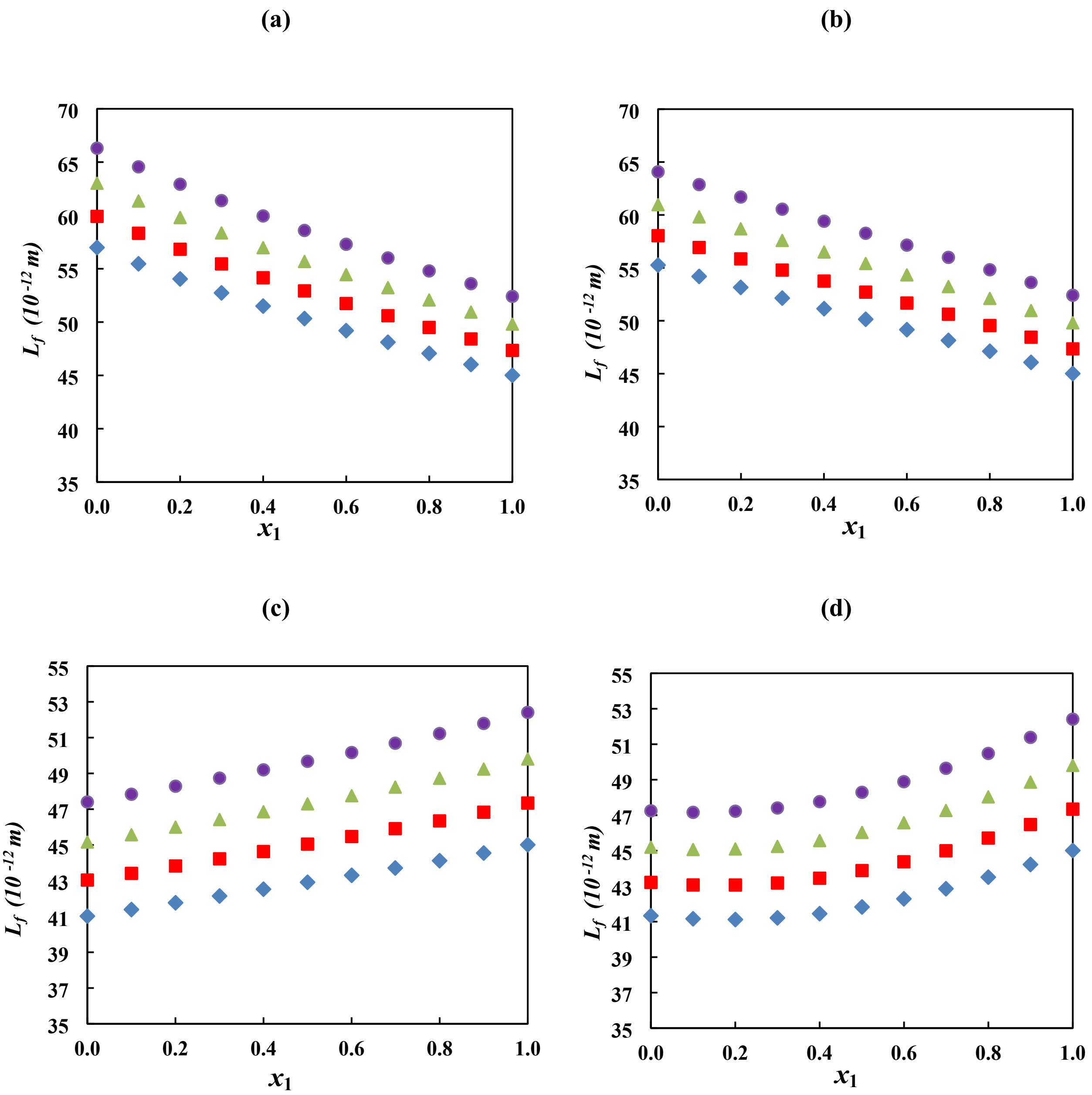

Fig. S5. Plot of intermolecular free length (L_f) for the binary mixtures: (a) {DMA + 1-butanol}, (b) {DMA+ 1-pentanol}, (c) {DMA+ FFL}and (d) {DMA+ FA} as function of the mole fraction of DMA at 293.15 K (◆), 303.15 K (■), 313.15 K (▲), 323.15 K (●).

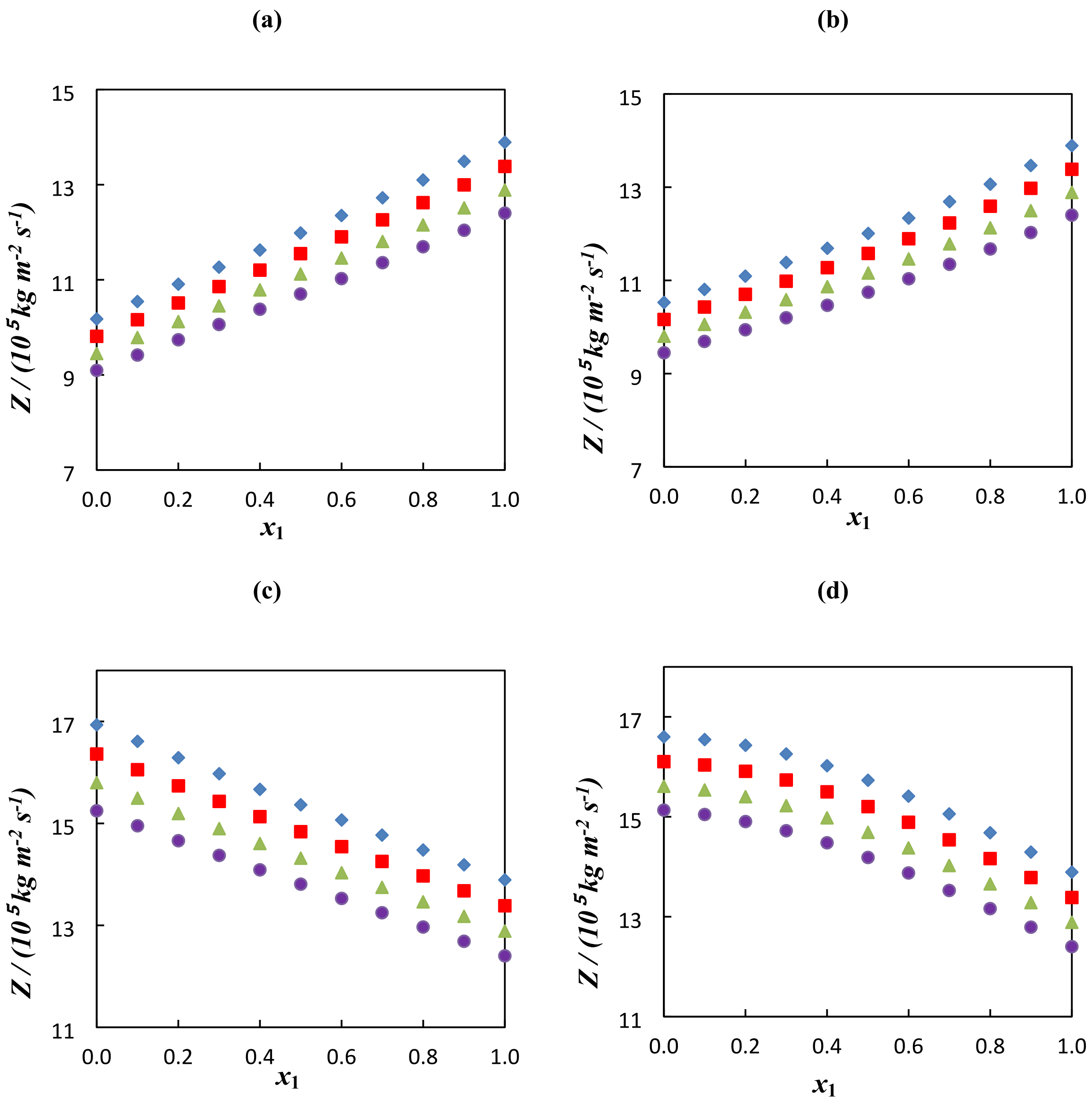

Fig. S6. Plot of specific acoustic impedance (Z) for the binary mixtures: (a) {DMA + 1-butanol}, (b) {DMA+ 1-pentanol}, (c) {DMA+ FFL} and (d) {DMA+ FA} as function of the mole fraction of DMA at 293.15 K (◆), 303.15 K (■), 313.15 K (▲), 323.15 K (●).

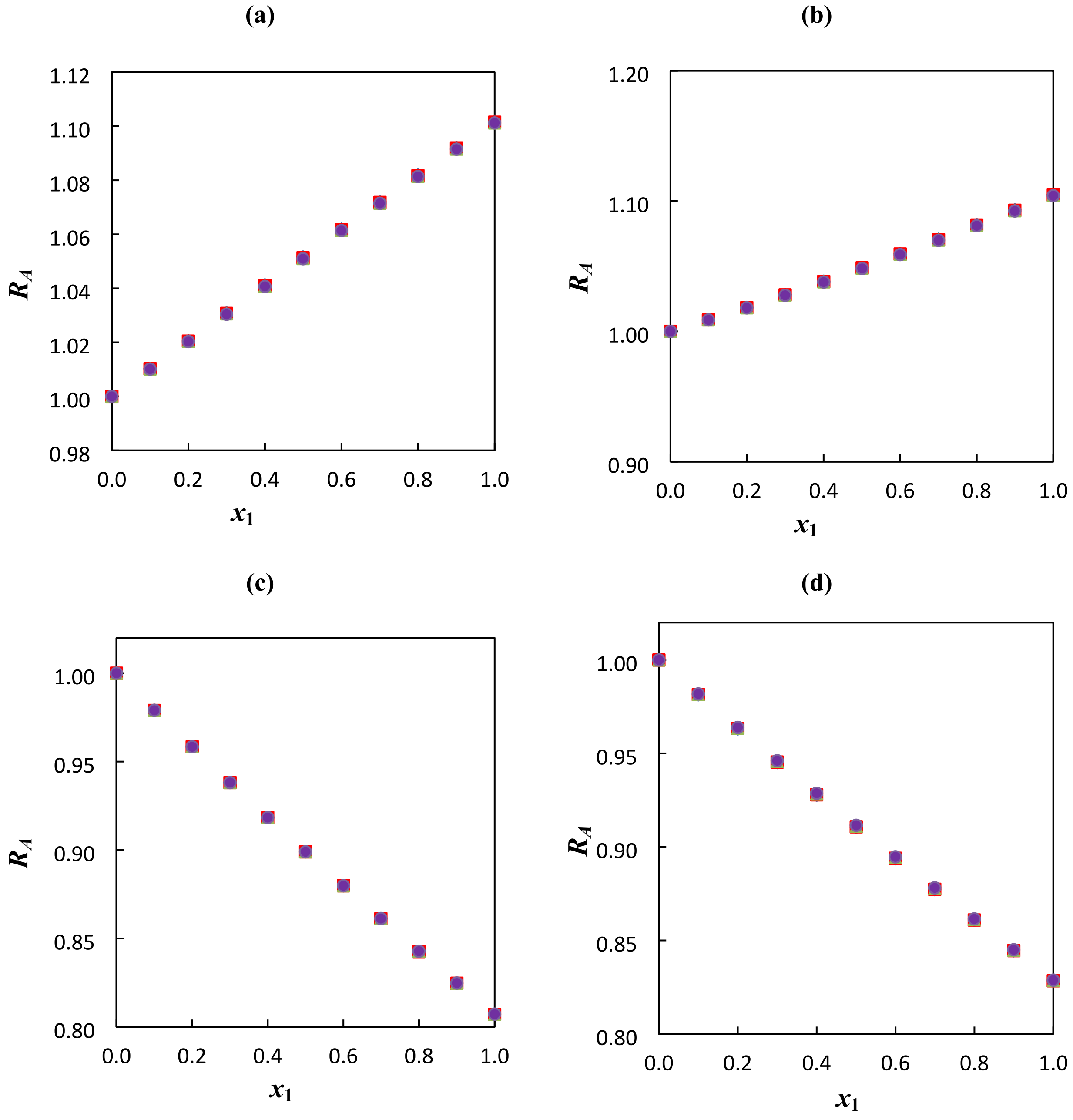

Fig. S7. Plot of relative association (R_A) for the binary mixtures: (a) {DMA + 1-butanol}, (b) {DMA+ 1-pentanol}, (c) {DMA+ FFL} and (d) {DMA+ FA} as function of the mole fraction of DMA at 293.15 K (◆), 303.15 K (■), 313.15 K (▲), 323.15 K (●).

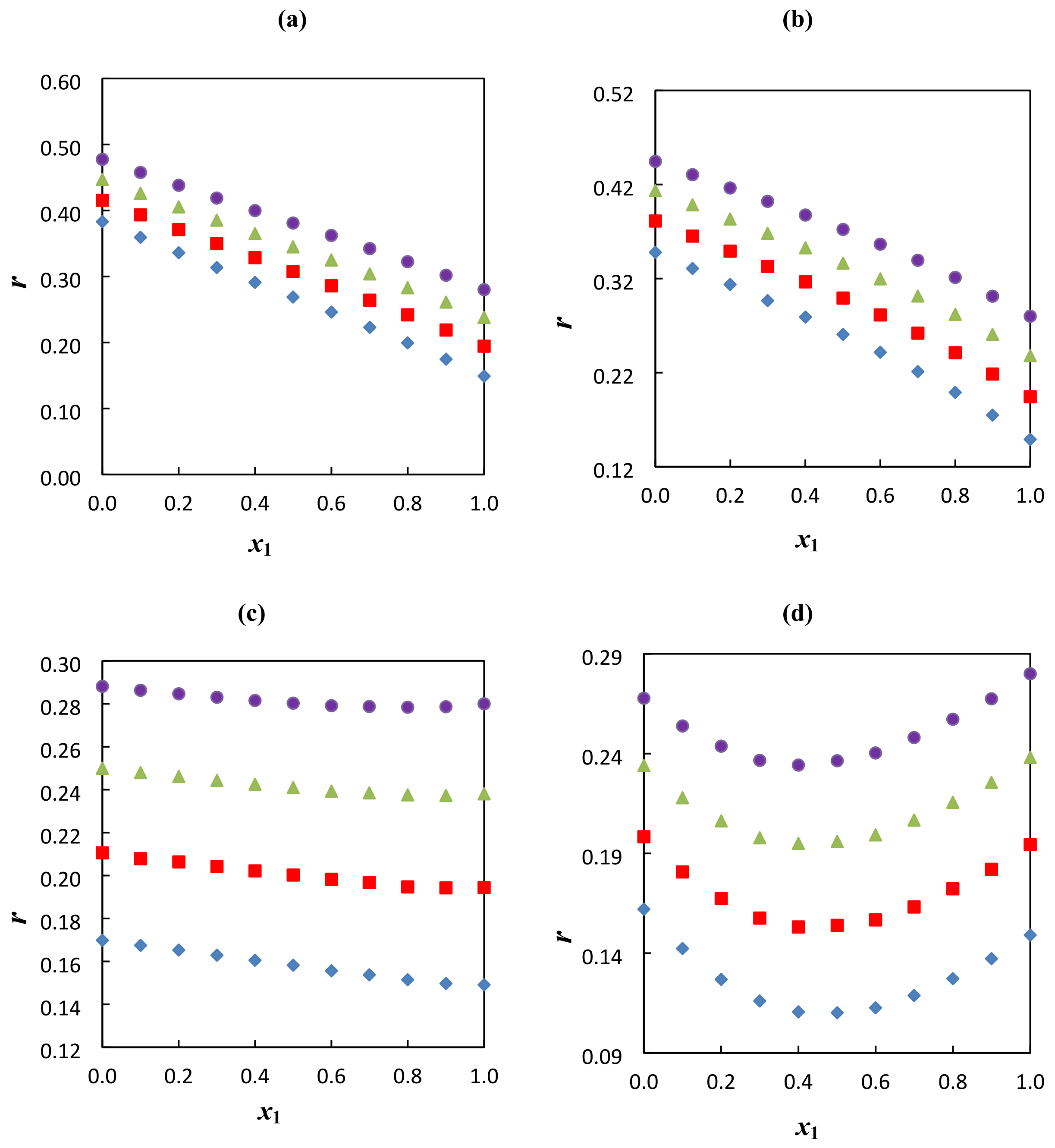

Fig. S8. Plot of relaxation strength (r) for the binary mixtures: (a) {DMA + 1-butanol}, (b) {DMA+ 1-pentanol}, (c) {DMA+ FFL} and (d) {DMA+ FA} as function of the mole fraction of DMA at 293.15 K (◆), 303.15 K (■), 313.15 K (▲), 323.15 K (●).

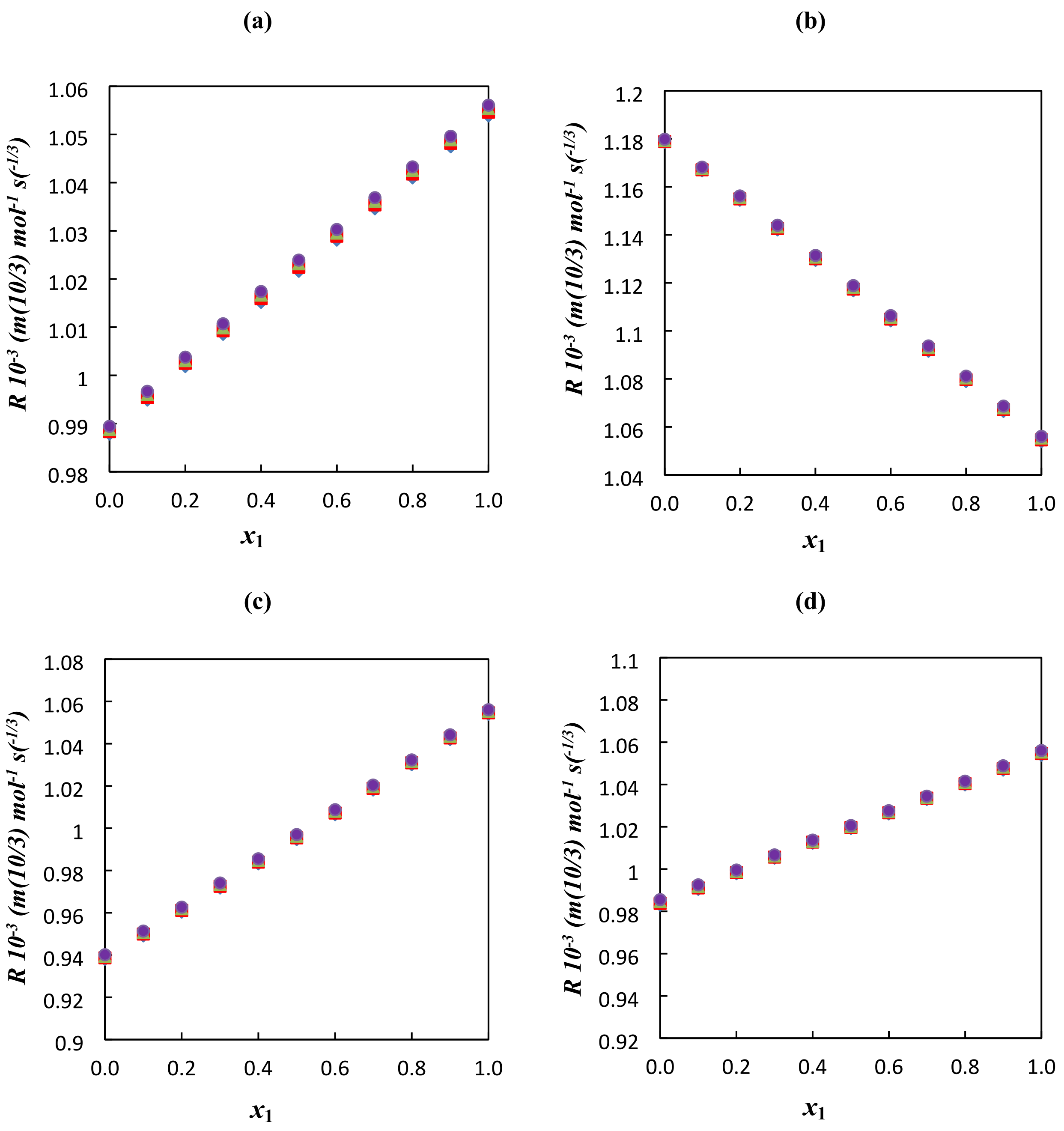

Fig. S9. Plot of Rao's molar sound function (R) for the binary mixtures: (a) {DMA + 1-butanol}, (b) {DMA+ 1-pentanol}, (c) {DMA+ FFL} and (d) {DMA+ FA} as function of the mole fraction of DMA at 293.15 K (◆), 303.15 K (■), 313.15 K (▲), 323.15 K (●).

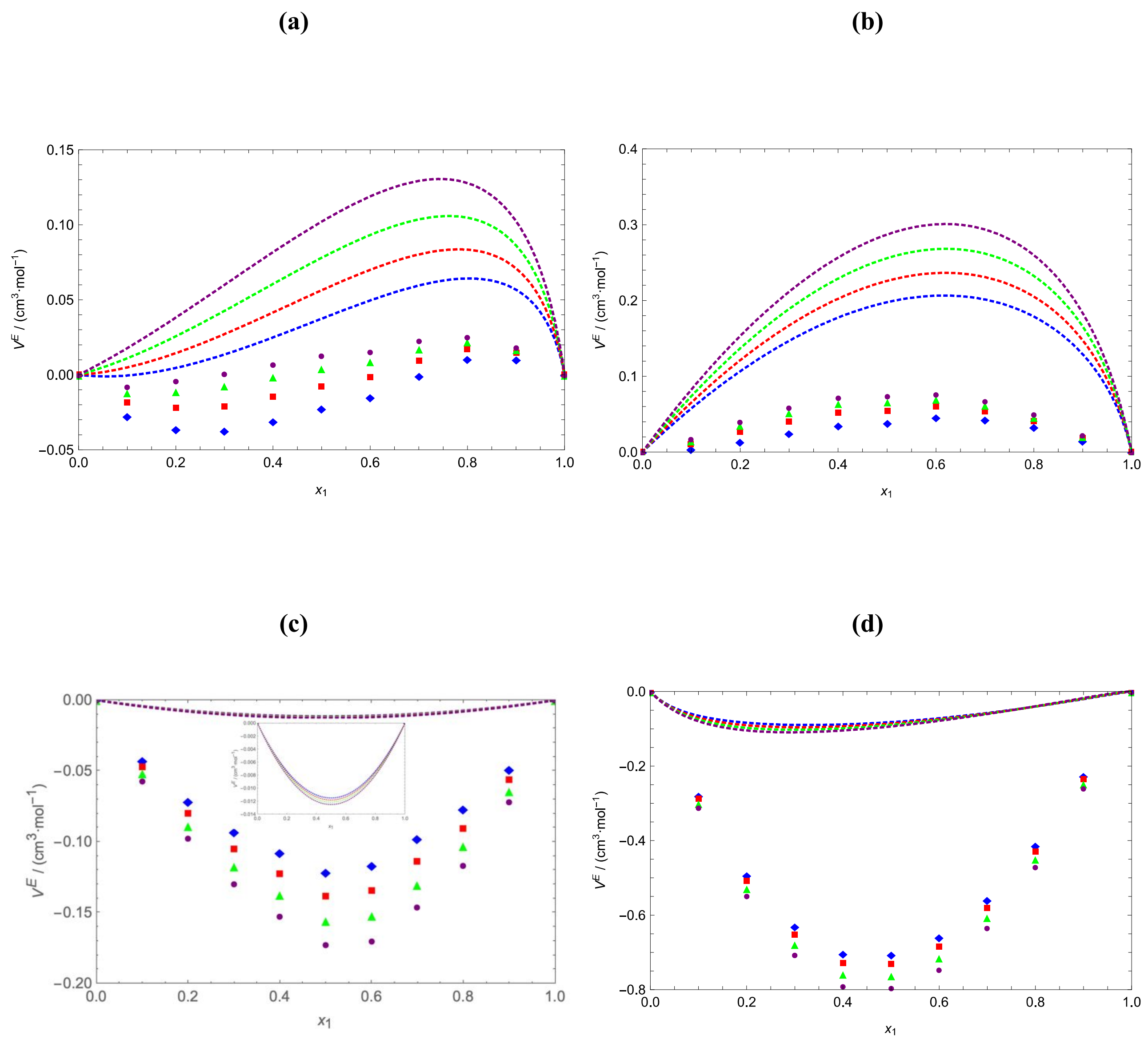

Fig. S10: Plot of excess molar volumes (V_m^E) for the binary systems; (a) DMA (1)+1-butanol (2), (b) DMA (1)+1-pentanol (2), (c) DMA (1) + FFL (2) and (d) DMA (1)+FA (2) as function of composition expressed in mole fraction at T = 293.15 K (◆), 303.15 K (■), 313.15 K (▲), 323.15 K (●). The lines were generated using PC-SAFT EoS.

Table S1: Excess molar volume, V_m^E, excess isentropic compressibility, k_s^E, and excess refractive index, n_D^E, for binary systems N, N-dimethylacetamide (*1*) + 1-butanol, or 1-pentanol, or furfural, or furfuryl alcohol (*2*) at (293.15, 303.15, 313.15 and 323.15) K and at pressure p = 0.1 MPa.

x_1	293.15 K			303.15 K			313.15 K			323.15 K		
	V_m^E/(cm^3 mol^{-1})	k_s^E (TPa^{-1})	n_D^E	V_m^E (cm^3 mol^{-1})	k_s^E (TPa^{-1})	n_D^E	V_m^E / (cm^3 mol^{-1})	k_s^E (TPa^{-1})	n_D^E	V_m^E / (cm^3 mol^{-1})	k_s^E (TPa^{-1})	n_D^E
**{N, N-dimethylacetamide (*1*) + 1-butanol (*2*)}**												
0	0.0000	0.00	0.0000	0.0000	0.00	0.0000	0.0000	0.00	0.0000	0.0000	0.00	0.0000
0.0998	-0.0269	-12.43	0.0004	-0.0180	-13.48	0.0004	-0.0111	-13.07	0.0003	-0.0071	-13.62	0.0003
0.2001	-0.0369	-20.75	0.0007	-0.0230	-21.88	0.0006	-0.0117	-21.78	0.0006	-0.0046	-22.69	0.0006
0.2997	-0.0376	-25.31	0.0008	-0.0216	-26.18	0.0007	-0.0075	-26.66	0.0007	0.0005	-28.30	0.0007
0.3999	-0.0308	-27.09	0.0008	-0.0147	-27.65	0.0008	-0.0011	-29.01	0.0006	0.0072	-30.21	0.0007
0.4995	-0.0227	-26.45	0.0008	-0.0082	-26.87	0.0007	0.0041	-28.39	0.0006	0.0127	-29.54	0.0007
0.5998	-0.0151	-24.04	0.0007	-0.0021	-24.20	0.0006	0.0089	-25.25	0.0005	0.0154	-26.26	0.0005
0.7004	-0.0011	-19.80	0.0006	0.0089	-19.79	0.0005	0.0170	-21.13	0.0005	0.0225	-21.93	0.0002
0.7996	0.0105	-14.28	0.0004	0.0170	-14.21	0.0003	0.0223	-15.24	0.0003	0.0254	-15.80	0.0002
0.9004	0.0098	-7.56	0.0002	0.0139	-7.24	0.0001	0.0165	-7.73	0.0001	0.0179	-8.05	0.0001
1.0000	0.0000	0.00	0	0.0000	0.00	0.0000	0.0000	0.00	0.0000	0.0000	0.00	0.0000
**{N, N-dimethylacetamide (*1*) + 1-pentanol (*2*)}**												
0	0.0000	0.00	0.0000	0.0000	0.00	0.0000	0.0000	0.00	0.0000	0.0000	0.00	0.0000
0.0991	0.0039	-6.95	0.0002	0.0100	-7.34	0.0001	0.0149	-6.71	0.0001	0.0174	-8.08	0.0001
0.1993	0.0141	-11.53	0.0003	0.0266	-12.25	0.0002	0.0361	-12.29	0.0002	0.0407	-13.62	0.0001
0.2994	0.0253	-14.41	0.0004	0.0403	-15.37	0.0003	0.0527	-15.89	0.0003	0.0595	-17.10	0.0002
0.4004	0.0347	-15.54	0.0004	0.0513	-16.59	0.0003	0.0642	-17.50	0.0003	0.0718	-18.53	0.0002
0.5007	0.0384	-15.62	0.0004	0.0539	-16.67	0.0003	0.0665	-17.88	0.0003	0.0741	-18.74	0.0002
0.6000	0.0458	-14.36	0.0003	0.0593	-15.41	0.0002	0.0701	-16.61	0.0002	0.0765	-17.28	0.0001

0.7002	0.0428	-12.23	0.0001	0.0534	-13.18	0.0001	0.0621	-14.35	0.0001	0.0674	-14.82	-0.0001
0.8000	0.0333	-9.15	0.0001	0.0406	-9.92	0.0001	0.0464	-10.87	-0.0001	0.0503	-11.15	-0.0001
0.8994	0.0147	-5.40	0.0001	0.0179	-5.86	0.0001	0.0206	-6.43	-0.0001	0.0224	-6.58	-0.0001
1.0000	0.0000	0.00	0.0000	0.0000	0.00	0.0000	0.0000	0.00	0.0000	0.0000	0.00	0.0000
**{N, N-dimethylacetamide (*1*) + furfural (*2*)}**												
0	0.0000	0.00	0.0000	0.0000	0.00	0.0000	0.0000	0.00	0.0000	0.0000	0.00	0.0000
0.1001	-0.0422	-1.98	0.0002	-0.0470	-2.74	0.0003	-0.0508	-2.99	0.0004	-0.0562	-3.54	0.0005
0.2002	-0.0722	-3.44	0.0004	-0.0809	-4.32	0.0006	-0.0893	-5.29	0.0007	-0.0979	-6.30	0.0008
0.3002	-0.0933	-4.59	0.0006	-0.1058	-5.79	0.0008	-0.1177	-7.09	0.0009	-0.1299	-8.46	0.0010
0.3999	-0.1078	-5.36	0.0007	-0.1231	-6.74	0.0008	-0.1376	-8.30	0.0009	-0.1525	-9.92	0.0010
0.4999	-0.1225	-5.72	0.0007	-0.1397	-7.23	0.0007	-0.1567	-8.92	0.0009	-0.1735	-10.68	0.0009
0.6002	-0.1169	-5.80	0.0007	-0.1350	-7.22	0.0008	-0.1521	-8.96	0.0008	-0.1700	-10.70	0.0009
0.6991	-0.0983	-5.10	0.0008	-0.1148	-6.44	0.0008	-0.1307	-7.91	0.0008	-0.1464	-9.46	0.0009
0.7998	-0.0770	-4.21	0.0006	-0.0909	-5.59	0.0007	-0.1030	-6.40	0.0007	-0.1165	-7.60	0.0007
0.9000	-0.0489	-2.65	0.0003	-0.0565	-3.24	0.0003	-0.0642	-3.90	0.0004	-0.0716	-4.63	0.0004
1.0000	0.0000	0.00	0.0000	0.0000	0.00	0.0000	0.0000	0.00	0.0000	0.0000	0.00	0.0000
**{N, N-dimethylacetamide (*1*) + furfuryl alcohol (*2*)}**												
0.0000	0.0000	0.00	0.0000	0.0000	0.00	0.0000	0.0000	0.00	0.0000	0.0000	0.00	0.0000
0.1000	-0.2796	-12.62	0.0016	-0.2892	-13.68	0.0017	-0.3000	-15.04	0.0017	-0.3112	-15.91	0.0019
0.2002	-0.4927	-22.32	0.0028	-0.5089	-24.10	0.0028	-0.5277	-26.32	0.0029	-0.5475	-28.18	0.0031
0.2999	-0.6309	-29.00	0.0035	-0.6537	-31.71	0.0036	-0.6786	-34.70	0.0037	-0.7065	-37.30	0.0039
0.4000	-0.7033	-32.45	0.0039	-0.7290	-35.66	0.0041	-0.7575	-38.80	0.0042	-0.7899	-42.21	0.0044
0.5004	-0.7063	-32.74	0.0039	-0.7325	-35.90	0.0041	-0.7624	-39.51	0.0042	-0.7952	-42.88	0.0044
0.5996	-0.6601	-30.85	0.0037	-0.6860	-34.16	0.0038	-0.7148	-37.74	0.0039	-0.7462	-41.06	0.0041
0.6994	-0.5590	-26.28	0.0031	-0.5809	-29.27	0.0032	-0.6056	-32.11	0.0033	-0.6332	-35.09	0.0035
0.7997	-0.4137	-19.53	0.0023	-0.4305	-21.68	0.0024	-0.4493	-24.10	0.0024	-0.4703	-26.44	0.0024
0.8997	-0.2269	-10.86	0.0013	-0.2369	-12.38	0.0013	-0.2476	-13.79	0.0013	-0.2596	-15.15	0.0013
1.0000	0.0000	0.00	0.0000	0.0000	0.00	0.0000	0.0000	0.00	0.0000	0.0000	0.00	0.0000

Standard uncertainties u are u(T) = 0.02 K, u(p) = 0.04 MPa, and the combined expanded uncertainties Uc in mole fraction, density, speed of sound, refractive index, excess molar volume, deviation in isentropic compressibility and deviation in refractive index were Uc(x) = ±0.0006, Uc(ρ) = 0.8 Kg.m^{-3}, Uc(c) = 2.81 m.s^{-1}, Uc(n_D) = 0.0007, Uc(V^E_m) = 0.003 cm^3·mol^{-1}, Uc (k^E_s) = 0.17 TPa−1 and Uc (n^E_D) = 0.0008, respectively, (0.95 level of confidence).
